# Revisiting potassium intercalation in graphite: an *operando* characterisation and computational approach

**DOI:** 10.1039/d5eb00184f

**Published:** 2025-11-27

**Authors:** Zhenyu Guo, Kang Wang, Yuanzhu Zhao, Gang Cheng, Yichen Huang, Connor Wright, Zonghao Shen, Hossein Yadegari, Jinglin Jiang, Fei Xie, Kaitian Zheng, Cecilia Mattevi, Carla Molteni, Peter D. Haynes, Mary P. Ryan, Maria-Magdalena Titirici

**Affiliations:** a Department of Chemical Engineering, Imperial College London London SW7 2AZ UK m.titirici@imperial.ac.uk; b Department of Materials, Imperial College London London SW7 2AZ UK; c Imperial Global Singapore (IGS), Imperial College London Singapore 138602 Singapore; d Key Laboratory for Renewable Energy, Beijing Key Laboratory for New Energy Materials and Devices, Beijing National Laboratory for Condensed Matter Physics, Institute of Physics, Chinese Academy of Sciences Beijing 100190 China; e Chemical Engineering Research Center, State Key Laboratory of Chemical Engineering, School of Chemical Engineering and Technology, Tianjin University Tianjin 300072 China; f Department of Physics, King's College London Strand London WC2R 2LS UK; g Advanced Institute for Materials Research (WPI-AIMR), Tohoku University 2-1-1 Katahira Aobaku Sendai Miyagi 980-8577 Japan

## Abstract

Potassium-ion batteries (KIBs) with graphite anodes are emerging as a highly promising “beyond lithium” technology driven by battery demands, potassium's abundant reserves and the inherent similarities in intercalation chemistry to lithium-ion systems. Despite this potential, a understanding of potassium intercalation into graphite, particularly concerning early intercalation stages and the in-plane ordering of K^+^ within graphite intercalation compounds (GICs), lacks sufficient elucidation. Herein, we employed a multi-modal, *operando* characterisation approach to elucidate the correlation of electrochemical potassiation and structural evolution in graphite, hence unravelling the specific mechanisms of K-ion storage. *Operando* electrochemical dilatometry precisely quantifies the macroscopic volume expansion of a graphite electrode during potassiation. Meanwhile, *operando* synchrotron X-ray diffraction (XRD) records ordered phase transitions during early-stage intercalation, detailing the formation of distinct GIC phases. Furthermore, Raman spectroscopy and density-functional theory (DFT) reveal the in-plane ordering of K^+^ within the graphite gallery and stacking modes. *Operando* optical microscope and UV-vis spectroscopy together provide insights into the changing optical properties, linking these changes to different GICs and electronic structural changes. This comprehensive study offers fundamental mechanistic insights into K-ion storage in graphite, paving the way for the rational design of high-performance KIB anodes.

## Abstract 2

A multi-modal, *operando* characterisation approach was used to revisit the correlation of electrochemical potassiation and structural evolution in graphite, hence unravelling the specific mechanisms of K-ion storage.

## Graphical abstract

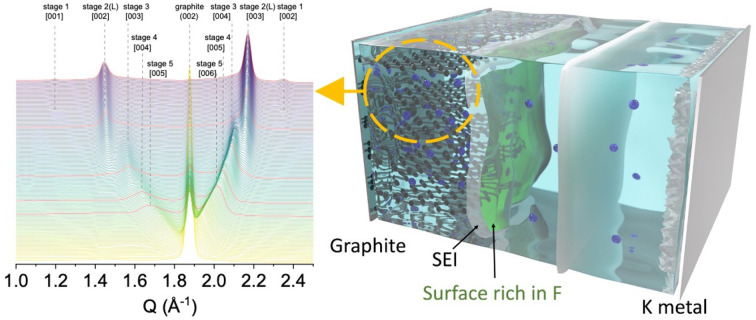

**Broader context**

The global push for sustainable energy solutions and grid-scale storage has underscored the limitations of lithium-ion batteries (LIBs), particularly concerning the cost and geopolitical constraints of related resources. This situation has driven intense research into “beyond lithium” technologies that use more abundant elements. Among these, sodium-ion batteries and potassium-ion batteries (KIBs) have emerged as highly promising and scalable alternatives. KIBs feature a similar intercalation chemistry to LIBs while benefiting from the vast and widely distributed reserves of potassium. A key challenge in the commercialisation of KIBs lies in the development of high-performance and long-lasting electrode materials. Carbonaceous materials, especially graphite, are the main candidates for KIB anodes due to their high gravimetric capacity and industrial maturity. However, the larger ionic radius of potassium leads to different intercalation dynamics compared to lithium, and a comprehensive, in-depth understanding of the real-time structural and electronic changes that occur during potassiation is critically needed to inform the rational design of next-generation KIBs. This work addresses this fundamental knowledge gap by investigating the intercalation mechanisms of potassium in graphite.

## 1. Introduction

Lithium-ion batteries (LIBs) significantly mitigate climate change by facilitating the transition from fossil fuels to electric transportation, provided the electricity used is generated from renewable sources. According to the European Commission and World Economic Forum, global battery demand is projected to surge fourteen-fold from 2018 to 2030, with the EU potentially accounting for 17% of that increase.^1^ Therefore, the increasing demand for critical metals used in LIBs is forecasted. To solve this dilemma, beyond-lithium technologies are of great significance to meet the rapidly expanding energy storage market.^2–5^

Potassium-ion batteries (KIBs) share similar intercalation chemistry to LIBs but with significant advantages.^6,7^ Potassium's global abundance (17 000 ppm *vs.* 18 ppm for Li),^8^ and even distribution directly translates into a substantial cost advantage (Li_2_Co_3_: $97 000 per ton, K_2_O: $980 per ton in 2022).^9^ Furthermore, the existing manufacturing infrastructure is readily adaptable for KIB production at a low cost, which is a critical factor for large-scale energy storage applications. Electrochemically, potassium exhibits a 0.09 V lower electrochemical potential than Li^+^/Li in a carbonate solvent.^6^ Also, K^+^ has the highest ion mobility and conductivity because its Stokes’ radius of 3.6 Å in propylene carbonate solvents is smaller than that of both Li^+^ (4.8 Å) and Na^+^ (4.6 Å).^10^ This smaller solvated size, despite a larger ionic radius (1.38 Å), enables faster diffusion of K^+^, allowing increased power density and superior low-temperature performance.^11,12^

Graphite, an outstanding anode material in commercial LIBs, is also a promising candidate for KIBs. The intercalation of K^+^ into the graphite lattice is *via* a well-established “staging” mechanism.^6,7,13–15^ Intercalating K^+^ between the layers of graphite results in the formation of different K-GICs with varied chemical, structural and electronic properties.^16^ K-GICs are characterised by a specific crystallographic arrangement of K^+^ within the van der Waals galleries of the graphite host and a defined number of graphene layers separating adjacent intercalated K^+^ layers. The maximum stable stoichiometry of the K-GIC is KC_8_ in a voltage window of 0–2.5 V *vs.* K^+^/K at room temperature. Hence, it has a high theoretical capacity of 279 mAh g^−1^.^8^ However, the reliance on mined natural graphite, often concentrated in a few geopolitical regions, presents environmental challenges and supply chain risks. Simultaneously, the production of synthetic graphite, typically derived from petroleum coke and coal tar pitch (by-products of the fossil fuel industry), also carries a substantial environmental footprint due to its energy-intensive graphitisation process (temperatures up to 3000 °C). There are also opportunities to produce graphite more sustainably to fuel the battery revolution, such as carbonisation of biomass in a more innovative method,^17,18^ and recycling graphite from the end-of-life batteries.^19,20^

In 2024, a startup GROUP1 launched the world's first K-ion 18 650 cylindrical batteries with graphite anode and a cell energy density of 160–180 Wh kg^−1^, benchmarking the current graphite//LiFPO_4_ cells.^21^ Despite the promising commercialisation efforts, the large ionic size of K^+^ introduces practical obstacles such as large volume changes, low initial coulombic efficiency, and limited cycle life. To overcome these challenges and enable the rational design of KIBs, it is crucial to gain a fundamental, in-depth understanding of the K^+^ storage mechanism within the graphite lattice.

The interaction between potassium or lithium with graphite can be traced back to the 1920s–1980s, when solid-state chemical synthesis and the physics of K-GICs and Li-GICs were focused.^22–25^ Graphite has dominated the anode market for LIBs since Sony commercialised LIBs in the 1990s.^26^ The breakthroughs of electrochemical intercalation of K^+^ into graphite at room temperature were reported in 2015 by Ji *et al.*, who ignited research interest in graphite anodes for KIBs.^7^ Since 2015, a decade has seen significant progress in unveiling the K-GIC staging mechanism. Yet, the precise staging sequence remains a subject of considerable debate in the literature. For example, Komaba *et al.* use *operando* XRD data and DFT calculations together to reveal the staging changes from graphite to stage 1 *via* disorderly high stages, stage 4L, stage 3L, stage 2L, and stage 1.^15^ Liu *et al.* use *operando* XRD to report the sequence *via* graphite, stage 5 (KC_60_), stage 4 (KC_48_), stage 3 (KC_36_), stage 2 (KC_24_/KC_16_), and stage 1 (KC_8_).^14^ DFT calculations suggest the formation energetics of K^+^ intercalation into graphite can be unfavourable at very low K^+^ concentrations and vary with the K^+^ content. The prevailing view is that the formation of high stages is highly disordered, a hypothesis largely based on the absence of well-resolved XRD peaks in the early intercalation phase.

This work presents a comprehensive, multi-modal investigation that provides a mechanistic pathway for K^+^ intercalation into graphite. We combine *operando* characterisation techniques, including synchrotron XRD and electrochemical dilatometry, with first-principles DFT calculations^27^ to precisely correlate structural changes at both the atomic and macroscopic levels (see SI section 1.7). DFT phonon calculations of the layer breathing modes (LBMs) of K-GICs are conducted based on XRD analysis and the low-frequency region from Raman spectra. Our findings not only resolve the conflicting staging sequences reported in the previous literature but also reveal a detailed pathway from liquid-like stages to ordered in-plane stages before reaching the final stage 1 K-GIC. Furthermore, we provide a detailed analysis of the electronic structure changes using an *operando* microscope and UV-Vis spectroscopy. These insights offer fundamental principles for designing high-performance KIB anodes.

## 2. Results and discussion

### 2.1. Electrochemistry

**Fig. 1:**
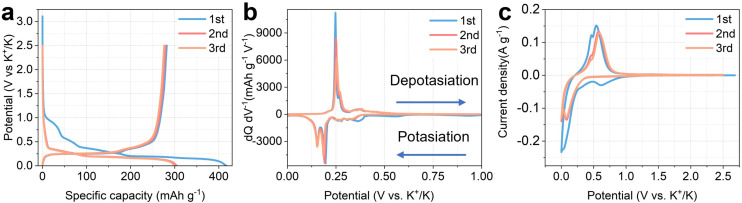
The electrochemistry of K metal|1 M KN(SO_2_F)_2_ in EC and DEC (1 : 1 in volume)|graphite batteries. (a) The first three galvanostatic discharge–charge cycles at 16 mA g^−1^; (b) the differential capacity of the first three galvanostatic discharge–charge cycles; (c) the first three cyclic voltammetry (CV) profiles at 0.1 mV s^−1^.

The electrochemical performance of graphite as an anode material for KIBs was characterised in a three-electrode configuration: K metal|1 M KN(SO_2_F)_2_ (KFSI) in ethylene carbonate (EC) and diethyl carbonate (DEC) (1 : 1 in volume) |graphite (Fig. 1a). Fig. S1 shows the characterisation of the graphite. This electrolyte composition was chosen due to several compelling advantages over alternatives. The 1 M KFSI in EC/DEC electrolyte offers stable electrochemical performance during cycling (Fig. S2 in SI), consistent with reported literature.^10,14,15^ Additionally, it was reported that this electrolyte system demonstrates a high initial coulombic efficiency (up to 89%) and higher ionic conductivity (10.7 mS cm^−1^).^28^ In contrast, the widely adopted slat KPF_6_-based electrolytes show lower initial coulombic efficiencies (50–60%) and limited solubility of up to 0.8 M in EC : DEC.^29^ A critical consideration for long-term cell stability is the interaction with current collectors. It is worth noting that the use of a pure KFSI electrolyte, free from chloride impurities, is expected to mitigate corrosion of the Al current collector commonly employed on both the anode and cathode sides, thereby ensuring the overall cell lifespan.^28,30^

All discharge (potassiation) and charge (depotassiation) profiles exhibit a sloping region at high potential and a long plateau capacity at low potential. The observed plateau regions are reflected as sharp peaks in the differential capacity plot shown in Fig. 1b. The electrochemical potentials associated with K^+^ intercalation into graphite determine the overall cell voltage and therefore energy density. This differential capacity plot indicates that K^+^ intercalation into graphite occurs at *ca.* 0.1–0.2 V *vs.* K^+^/K. This value is slightly higher than those observed for the Li case (*ca.* 0.1 V *vs.* Li^+^/Li), which suggests a lower likelihood of K metal plating. Cyclic voltammetry (CV) was conducted at a scan rate of 0.1 mV s^−1^ (Fig. 1c) for the first three cycles. The first cycle features an increased CV area attributed to solid electrolyte interphase (SEI) formation, in line with a higher discharge (potassiation) capacity. It is widely accepted that the formation of an SEI is the main reason for the irreversible loss in KIBs, which will be discussed in section 2.5.

Regarding the notation, the stage index *n* is extensively employed to describe the periodic stacking of K-GIC. The stage index, denoted as *n* (an integer: 1, 2, 3, *etc*.), describes the number of graphite layers between two adjacent intercalated potassium layers. Diffraction patterns play a crucial role in identifying these staged structures. The stage diffraction [00*n*] peak serves as a definitive indicator of staging behaviour in K-GICs, indicating the periodic repetition of intercalant layers along the crystallographic *c*-axis. It is important to clarify the [00*n*] notation: while Miller indices (*hkl*) incorporate the in-plane ordering of the potassium layers, it instead functions as a classification system specifically tailored for describing the characteristic layering patterns observed in GICs. In contrast to the macroscopic *c*-axis staging described by the stage index and [00*n*] stage diffraction, Miller indices (*hkl*) are utilised to consider the in-plane ordering of the potassium layers. This distinction is crucial for a complete structural description. For example, in stage 1 KC_8_, the Miller index (00*l*) quadruples relative to the stage diffraction [001] due to a difference in-plane ordering is considered.

Furthermore, the in-plane potassium ordering is denoted using the notation *p*(*A* × *B*)*Rφ*, where *p* stands for primitive, *A* and *B* are the in-plane lattice parameters, and *φ* defines the rotational angle between the intercalated lattice and the graphite cell vectors. For example, *p*(2 × 2)*R*0° means this unit cell is twice as long as the graphite unit cell along both of its principal axes in the plane, and the potassium superlattice is perfectly aligned (no rotation) with the underlying graphite lattice. This detailed notation allows for a precise characterisation of the potassiation of graphite.

### 2.2. Electrode-level changes *via operando* dilatometry

**Fig. 2:**
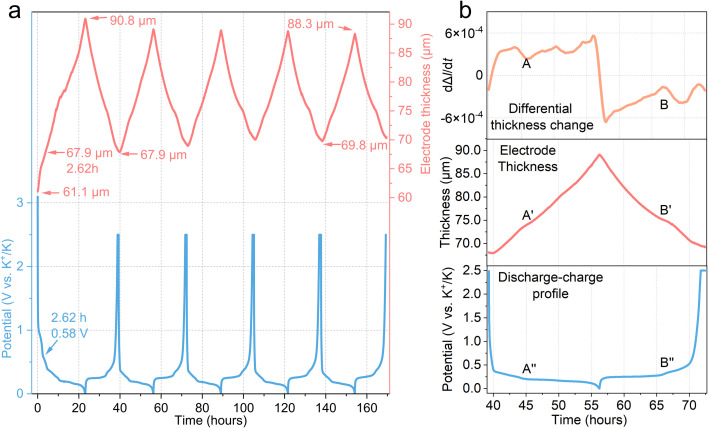
*Operando* electrochemical dilatometry analysis. (a) For the first five cycles (in blue) at 16 mA g^−1^, with the change in thickness (in pink). The thickness of the pristine graphite electrode is 61.1 μm; (b) the change of the electrode thickness and its differential change in the second cycle.

Understanding the dynamic volume changes of electrode materials during cycling is crucial for designing long-lasting KIBs. *Operando* electrochemical dilatometry, as shown in Fig. 2a, provides the periodic thickness variations of a graphite electrode in the first five discharge–charge cycles at 16 mA g^−1^. The pristine graphite electrode measures 61.1 μm in thickness (after calendaring). During the first potassiation cycle, the graphite electrode experienced a significant dilation of 29.7 μm (from 61.1 μm to 90.8 μm), which accounted for 48.6% of the thickness of the pristine graphite, representing the most pronounced expansion observed across all five cycles. This initial expansion is a critical behaviour of the first potassiation process. Following the first de-potassiation cycle, the electrode partially contracts to 67.9 μm, which is still equivalent to 110.3% of the pristine graphite thickness. The initial expansion of 6.8 μm, between OCV (61.1 μm) and 0.58 V (67.9 μm) in the first cycle, is primarily attributed to the formation of the solid electrolyte interphase (SEI) layer and irreversible potassium,^31^ which is in line with the CV analysis above. Apart from the SEI formation, the irreversible expansion after the first cycle is mainly attributed to irreversible potassiation,^6,32^ which is discussed in the *operando* microscope analysis (in section 2.4). For context, the theoretical *c*-axis expansion from pristine graphite (3.35 Å) to fully potassiated graphite KC_8_ (5.35 Å) is 60%. In comparison, a Li||graphite cell exhibits a much smaller initial thickness dilation. It is reported that a full lithiation causes only *ca.* 8.8% thickness dilation, including an irreversible thickness change of *ca.* 3.5%, while the theoretical *c*-axis expansion is *ca.* 10.4% from 3.35 Å (graphite) to *ca.* 3.70 Å (stage 1 Li-GICs, LiC_6_).^33^ This comparison highlights the significantly larger volume changes by K^+^ intercalation compared to lithium.

Following each depotassiation, a constant voltage of 2.5 V was applied until the current decreased to 1.6 mA g^−1^ to remove as much K^+^ as possible before a new potassiation cycle. In the first five cycles, a clear trend emerges that the thickness of the graphite electrode at the fully potassiated state gradually decreases, while, conversely, it gradually increases at the fully depotassiated state. In the fifth cycle, the full potassiation only causes an expansion of 18.5 μm from 69.8 μm to 88.3 μm. This suggests that the graphite parties rearrange to maintain a stable structure over cycles.

Fig. 2b provides a detailed investigation of the thickness change during the 2nd cycle, alongside its time derivative (dΔ*L*/d*t*), which directly represents the rate of thickness change. Analysing dΔ*L*/d*t* reveals two key points where the absolute value of the rate of thickness change reaches a minimum, indicating the slowest rates of dimensional variation. These minima occur around the 46th hour (point A) and the 67th hour (point B). Based on the corresponding electrochemical profiles, these minimal changes occur after the formation of stage 3 KC_24_. We hypothesise that a “liquid-like” stage 2L occurs, leading to the small plateau in the thickness rate between stage 3 KC_24_ and stage 2 KC_16_. From the literature, *operando* pressure measurements and electrochemical dilatometry reveal that Li||graphite cells exhibit a small pressure and thickness change during the transition between stage 3 and stage 2.^31,34^ This feature of minimal changes observed in our K||graphite system suggests that the stage 2L observed in Li-GICs also occurs in potassium intercalation.

A more rapid and notably linear correlation between thickness change and capacity is observed at the end of potassiation and the beginning of depotassiation (formation of stage 1 K-GIC), around the 56th hour in Fig. 2b. This fastest change in electrode thickness is primarily attributed to the dilation/contraction of the *c*-axis of graphite during the phase transition between stage 2 (average *d*-spacing = 4.35 Å) and stage 1 (average *d*-spacing = 5.35 Å). This stage transition involves a larger change in the average *d*-spacing than any other stage transitions in K-GICs. The observed linear change can be further elucidated by considering an “accordion-like” mechanism of intercalation, where not all K^+^ intercalate into each empty graphene interlayer but instead progressively move the graphite layers apart.^35^

### 2.3. Atomic expansion *via* synchrotron X-ray diffraction

To gain atomic-level insights into the expansion behaviour of the graphite anode during electrochemical potassiation, we conducted *operando* synchrotron X-ray diffraction (XRD) at Diamond beamline I15-1. For these measurements, we used a Diamond Radial *In situ* X-ray (DRIX) cell.^36^ The DRIX cell is custom-designed for *in situ* and *operando* experiments. In this experiment, the pristine graphite is potassiated under an extremely low current of 6.5 mA g^−1^ to ensure the early stages are captured. The potassiation process was stopped until the stage 1 K-GIC is partially formed, evident by the co-existence of characteristic diffraction peaks for both stage 1 KC_8_ and stage 2 KC_16_.

**Fig. 3:**
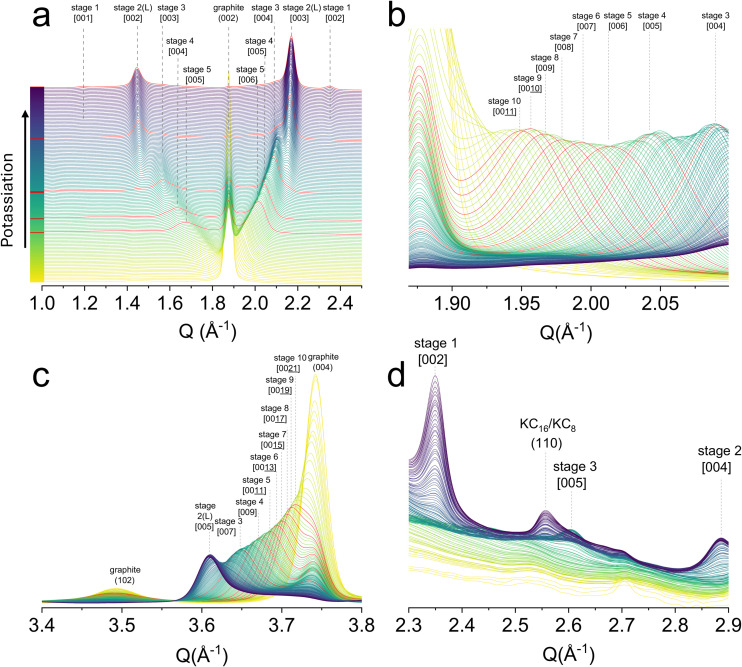
*Operando* synchrotron X-ray diffraction patterns for the first potassiation cycle (a) *Q* range from 1.0–2.5 Å^−1^, the highlighted red patterns are K-GICs stage 2–5 and stage 1/stage 2, stage 2L exists between stage 3 and stage 2; (b) *Q* range from 1.85 to 2.2 Å^−1^; (c) *Q* range from 3.4 to 3.8 Å^−1^; (d) *Q* range for the formation of [002] and [003] of stage 2 KC_16_.

At the very early stages of potassiation, when K^+^ concentrations are low, K^+^ intercalation into graphite is known to be energetically unfavourable.^37,38^ However, with potassiation proceeds, the initial intercalation events induce a crystallographic expansion along the *c*-axis. This expansion can lower the energy barrier for subsequent intercalation.^39^Fig. 3a illustrates this process that the pristine graphite (002) peak initially observed at *Q* = 1.878 Å^−1^ (*d*_interlayer_ = 3.35 Å) and then splits into two new peaks: (1) one located appears in high *Q* region (larger than 1.878 Å^−1^), which is associated with [00(*n* + 1)] stage diffraction of higher-stage K-GICs; (2) [00*n*] stage diffraction appears in the lower *Q* region (smaller than 1.878 Å^−1^). Indeed, the intercalation of K^+^ opens the *d*-spacing of two graphene layers to *ca.* 5.35 Å, clearly confirmed by the appearance of the characteristic KC_8_'s peak at 1.175 Å, while the *d*-spacing of the empty graphene layers (those not yet intercalated) largely remains at 3.35 Å. This observation aligns well with established literature on K-GICs.^6,7,14,15^ Based on these structural observations, the *c*-axis periodicity of the K-GICs can be described mathematically. The total distance along the *c*-axis (*d*_c_) of the minimal repeating unit in a stage *n* K-GIC can be calculated as follows:*d*_c_ = *d*_K layer_ × (*n* − 1) + *d*_graphite_where: *d*_K layer_ is the expanded interlayer by potassium (5.35 Å); *n* is the stage number, *d*_graphite_ is the spacing of pristine graphite layers (3.35 Å).

The average *d*-spacing of the stage *n* is denoted as *d*_00*n*_, which is calculated as follows:
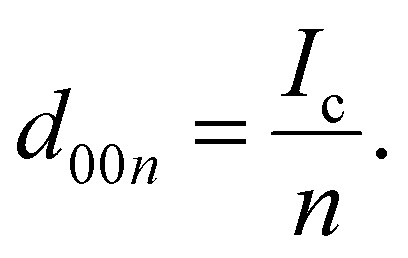


And the relationship between the *d*_00*n*_ and *d*_00*n*+1_ is as follows:
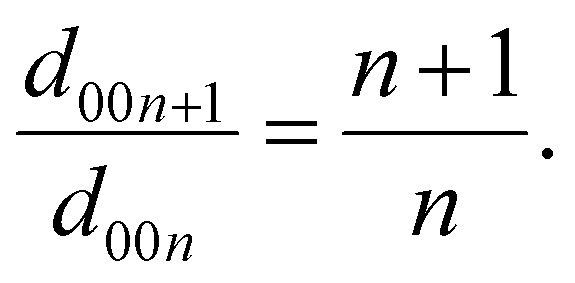


For example, the *d*_c_, *d*_001_, *d*_002_ and *d*_003_ of the stage 1 K-GIC is 5.35 Å, 5.35 Å, 2.68 Å and 1.78 Å, respectively. Similarly, the *d*_c_, *d*_001_, *d*_002_ and *d*_003_ of the stage 2 K-GIC is 8.70 Å, 8.70 Å, 4.35 Å and 2.90 Å, respectively.

DFT calculations suggest that significant local curvature in the graphene layers is induced during early intercalation.^38^ Localised curvature can disrupt the long-range planar order of graphite, leading to a noticeable decrease in the intensity of the pristine graphite (002) diffraction peak. Furthermore, compared with the rigid Rüdorff–Hofmann theory for ion intercalation,^40^ the Daumas–Hérold domain model offers a more nuanced and widely accepted picture for K-GICs.^15^ This model suggests that K-GICs can consist of many microscopic, localised domains, even in the same stage, but with the intercalants in different domains occupying different interlayer galleries. Instead of maintaining long-range order, localised structural deformations occur, transitioning to short-range order, as further confirmed by *operando* XRD and *ex situ* transmission electron microscopy (TEM) images (in section 2.5). The structural instability is further exacerbated by the gradual leaching of K^+^ from the graphite lattice, which contributes to the observed weakening of the graphite (002) peak's intensity. According to the Scherrer equation, smaller coherent scattering domains lead to broader diffraction peaks, consistent with the observed characteristics. A detailed analysis of these structural transformations, including *c*-axis stacking and *ab*-plane ordering, will be discussed in section 2.5.

#### *c*-Axis stacking

The staging behaviour of K-GICs during early intercalation remains a topic of considerable interest. Our synchrotron *operando* XRD reveals an ordered transition from graphite to high-stage K-GICs to low-stage K-GICs (see Fig. 3a). The *Q* range was selected from 1 to 2.5 Å^−1^, where all the [00*n*] and [00(*n* + 1)] stage diffractions of stage *n* are concentrated. More importantly, a crucial observation from the data is that the [00(*n* + 1)] diffraction peak of a given stage *n* consistently exhibits higher intensity and is better resolved than its [00*n*] counterpart across all observed stages. This phenomenon agrees well with the theory by Leung *et al.*, which suggests the maximum peak intensity in GICs occurs at [00(*n* + *m*)] peak, where *m* is 0 and 1 for Li-GICs and K-GICs, respectively.^41^ These differences result from the size of different intercalated ions and the structure factor of X-rays. Consequently, we have chosen to focus on the well-resolved [00(*n* + 1)] peaks for studying the structural evolution of K-GICs, especially for high stages.

To our best knowledge, all previous literature using *operando* XRD shows a broader peak with a low intensity for high stages during early intercalation, regardless of whether [00*n*] or [00(*n* + 1)] peaks were studied. This limitation may stem from the long exposure times of lab-based XRD systems, which are not powerful in distinguishing the rapid, dynamic changes occurring in the early stages of intercalation. The inherent advantages of synchrotron XRD, including its high flux, low background noise, and short data acquisition times, are instrumental in effectively capturing the rapid evolution of these early-stage phases in our study. From the XRD pattern in Fig. 3b, the [00(*n* + 1)] peaks of K-GICs progressively shift to higher *Q* values as more K^+^ is intercalated. Within these K-GICs stages, the continuous shift of peaks towards a higher *Q* range means that a single-phase, ordered reaction is predominantly taking place. Notably, the [00(11)] corresponding to stage 10 appears as the first well-resolved phase. The regions between graphite and stage 10 present those stages that have not yet intercalated or are in very dilute stages, maintaining a graphite-like spacing.

Peak fitting of the XRD patterns was conducted using the Voigt function from stage 10 to stage 5, and their intensity and full width at half maximum (FWHM) are summarised in Fig. S3 (SI). The FWHM of the [00(*n* + 1)] peaks remained consistent, ranging between 0.071 A^−1^ to 0.084 A^−1^ from stage 10 to stage 5, importantly, no sudden fluctuations in intensity and FWHM were observed. Instead, in the event that multiple high K-stages were coexisting prominently during the early stages, a massive increase in the FWHM of the peaks would be anticipated. The absence of such a broadening feature further supports a relatively smooth, ordered stage evolution. Additionally, the order transition from graphite's (004) peak to stage diffraction [00(2*n* + 1)] was also identified (Fig. 3c), complementing the evidence of the smooth phase change in the high stages. However, it must be noted that stage coexistence cannot be entirely avoided due to the relatively low K diffusivity within the solid graphite electrode,^38^ particularly in thick samples (>120 μm) where voltage hysteresis is ubiquitous. Our findings, different from all previous literature, suggest a more ordered early-stage intercalation process compared to previously obtained results with lab-based XRD techniques.

Apart from the early stages, the existence of stage 2L, a proposed partially intercalated variant of stage 2, remains arguable in K-GICs. While Liu *et al.* proved a metastable stage 2L K-GICs using Raman spectroscopy.^14^*Operando* microscope analysis (details in section 2.4) is in line with this phenomenon, which visualises the coexistence of phases (*e.g.*, stage 2 (orange) and stage 3 (blue), or stage 2 (orange) and stage 1 (gold)) during these two-phase reactions. In Li-ion cases, Fujimoto *et al.* proved the phases of stage 2 and stage 2L by two different *d*-spacings according to their XRD patterns.^42^ In our XRD patterns, due to the inherent wider peaks in K-ion systems, Fig. S4a and Fig. 3a reveal two continuously shifting and intensifying [002] and [003] peaks of stage 2 from stage 3, suggesting the changes of the *d*-spacing during the formation of stage 2. Together with the *operando* dilatometry discussed above, the electrode thickness evolves nonlinearly during potassiation, suggesting there is a stage 2L phase during potassium intercalation.

#### *c*-Axis stacking mode

Regarding the stacking mode during potassiation, the graphite (see its XRD pattern in Fig. S5) features an ABAB stacking sequence, evidenced by the characteristic graphite (101) and (102) peaks. Upon intercalation, forming high-stage GICs, the non-intercalated graphene layers largely maintain their original ABAB stacking sequence, while the regions directly sandwiching the potassium layers may adopt an AA stacking. This transition is quantitatively reflected by the decreasing intensity of the graphite (102) peak in the XRD patterns (Fig. 3c). As the K^+^ concentration increases, the transformation from ABAB to AA stacking within the intercalated regions is progressively completed, with the graphite (102) peak completely disappearing once stage 2 is fully reached. According to the theoretical study, the ABAB stacking mode is beneficial for initial intercalation of K^+^ by lowering the intercalation energy.^38^ This *c*-axis stacking behaviour, to our best knowledge, is reported for the first time in the K-GICs using experimental methods.

#### In-plane ordering of K^+^

The in-plane ordering of K^+^ within the graphite matrix is considerably more challenging to detect compared to the *c*-axis stacking, primarily due to the preferred crystallographic orientation of graphite and the inherently low X-ray scattering intensity of potassium. Moreover, temperature is a key factor that influences the in-plane K ordering within the graphite galleries.^43^ Literature suggests some possible in-plane arrangements for K-GICs: *p*(2 × 2)*R*0°, *p*(√7 × √7)*R*19.11°, *p*(3 × 3)*R*0°, *etc*.^15^

Fig. 3d shows the formation of an in-plane (*hk*0) peak of K-GICs located at *ca*. 2.55 Å^−1^. This peak initially appears as a broad feature when stage 2 K-GICs begin to form and becomes more visible and intense with the formation of stage 1 K-GICs (peak located at ∼2.35 A^−1^). This peak is assigned to the (110) in-plane reflection of KC_16_ and KC_8_. The presence of this (110) reflection suggests a *p*(2 × 2)*R*0° (as shown in Fig. S6 in SI) in-plane ordering of K^+^ within the stage 1 and stage 2 K-GICs. This in-plane ordering is firstly experimentally observed in the K-GICs. Additionally, the *p*(2 × 2)*R*0° in-plane ordering is consistent with the stoichiometry of KC_16_ of the stage 2, whereas a *p*(√7 × √7)*R*19.11° ordering would be more indicative of KC_24_. Similarly, the (120) in-plane reflection was identified at *ca.* 3.88 Å^−1^, in complement to (110) as evidence for in-plane ordering of KC_16_ and KC_8_ (Fig. S7 in SI). For other stages, however, the in-plane ordering remains unclear from our *operando* XRD patterns as shown in Fig. S3b (SI).

To further investigate these intricate in-plane changes, pair distribution function (PDF) analysis was employed. As shown in Fig. S8 (SI), peaks in the PDF predominantly arise from the nearest-neighbour distances of carbons within the K-GICs. The intercalation of K^+^ induces a slight expansion of the carbon matrix: the 6-membered carbon ring's C–C bond distances increase from 1.426 Å and 2.460 Å to 1.435 Å (+0.58%) and 2.469 Å (+0.38%), respectively, indicating minimal lattice distortion of the graphene sheets themselves. In contrast, K–K correlations exhibit inconsistent behaviour. A broad peak near 5.0 Å shifts leftward (from 5.0327 Å to 5.0279 Å) happens especially at stages 2 and 1, despite carbon matrix expansion, which is intuitively inconsistent with lattice expansion. This shift is in alignment with the *p*(2 × 2)*R*0° in-plane ordering of K^+^, which is predicted to have a nearest K–K distance of 4.92 Å. These PDF analyses support our XRD findings and are further supported by DFT calculations in section 2.3, which confirm that the *p*(2 × 2)*R*0° in-plane configuration dominates across stages 1–3 in K-GICs.

While specific in-plane ordering has been identified for stage 3, stage 2, and stage 1, it is important to note that if a stage only exhibits ordering along the *c*-axis without well-defined in-plane order, it should be designated as a ‘liquid-like’ (L) stage. Therefore, the overall potassiation of pristine graphite to form stage-1 KC_8_ can be summarised as a sequential process: it proceeds sequentially through liquid-like stages (stage 10L → 9L → ⋯ → 4L) followed by stage 3, stage 2L, and stage 2, before reaching the final stage 1.

### 2.4. *Operando* Raman spectroscopy

Raman spectroscopy is effective in distinguishing between different K-GICs based on their unique electronic structures, which exhibit Raman-active characteristics.^44^ Yadegari *et al.* use Raman spectroscopy with DFT calculations to reveal the stacking and in-plane structure of Li-GICs.^45^ We further extended the same protocol to KIBs, using high-resolution Raman spectroscopy supported by DFT calculations.

**Fig. 4:**
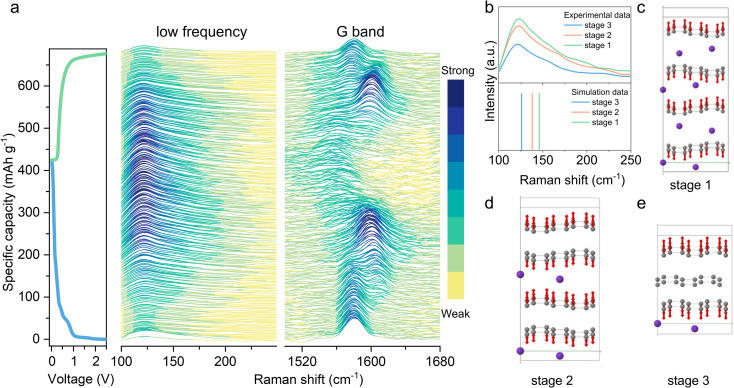
(a) *Operando* Raman spectra coloured according to their intensity with the first discharge–charge profile at a constant current of 16 mA g^−1^; (b) the experimental and simulated data of the low-frequency regions for different K-GICs; (c) the corresponding displacement patterns for the Raman-active modes.

Fig. 4a and Fig. S9 (in SI) show Raman spectra of the low-frequency region and the G band during the first discharge–charge cycle. In graphite, the G band (E_2g2_ mode) is one of the most prominent features originating from in-plane sp^2^ vibrations in rings or chains. From OCV to *ca.* 0.55 V, the G band remains nearly unchanged due to SEI formation. Followed by the widening of the G band, which suggests the early formation of K-GICs.^46^ The lower wavenumber peak (E_2g2_^(i)^) at *ca.* 1582 cm^−1^, originating from the uncharged graphene intralayer, decreases progressively.^47^ Whereas the emerging upper-frequency peak (E_2g2_^(b)^) at *ca.* 1598 cm^−1^, derived from the charged graphene layers (or boundary layer) adjacent to the K^+^ intercalant, grows gradually. The E_2g2_^(b)^ band blue shifts to a higher frequency due to an increase in the C–C bond force constants.^48^ The different intensity ratios of E_2g2_^(b)^ and E_2g2_^(i)^ bands can be used to determine the staging index of K-GICs.^49^ After the peak split, the stage 2 GIC forms as evident by the E_2g2_^(b)^ band reaching its highest intensity without the presence of the E_2g2_^(i)^ band.^45^ During the stage transition from stage 2 to stage 1, the E_2g2_^(b)^ band slowly diminishes as a result of the formation of superconductive stage 1 KC_8_, which reduces the optical skin depth and Raman scattering intensity.^50^

The low-frequency region (Fig. 4a, b) observed during the charge–discharge cycles arises from in-plane displacements (shear mode) and out-of-plane displacements (layer breathing modes) along the *c*-axis.^45^ The layer breathing modes (LBMs) are highly sensitive to both weaker van der Waals interactions, and the expansion of graphite interlayers is induced by intercalated species. This can be attributed to the appearance of a low-frequency region as the result of local strain-induced disorder, displacement, shear modes, and inner stress within the graphite lattice.^51,52^ Typically, the signal of the low-frequency region is weak or negligible from pristine graphite electrodes. However, after K^+^ intercalation, its intensity becomes comparable with that of the G and 2D bands, which is attributed to the greater interlayer separation. Previous Raman analysis of K-GICs focused on in-plane vibrational modes (G and 2D bands), and to the best of our knowledge, our research is the first to report on the low-frequency region in KIBs. Fig. S10 and Fig. S11 (SI) show the second and third cycles with *operando* Raman spectra, supporting the reproducibility and analysis discussed above.

DFT calculations were carried out to understand the K^+^ intercalation chemistry based on the low-frequency Raman spectra. There are more than ten Raman active modes in the high-frequency range of 1200–1650 cm^−1^. These modes are, in general, vibrations of in-plane C atoms with K^+^ remaining stationary. Due to large anharmonic effects, standard DFT calculations cannot offer accurate phonon frequencies for high-frequency Raman vibrational modes.^53–55^ Previous DFT work calculated the energy surface of 30 structures for various stages to find the most stable structures.^7,15^ Based on those structures, our DFT calculations show that K-GICs exhibit Raman-active modes in the low-frequency region. We calculated the phonon spectrum for relaxed cells of various stages to find mechanically stable stacking sequences. Our phonon calculations predicted that the stable sequences are AαAβAγAδ stacking for stage 1, AAα stacking for stage 2, and ABAα stacking for stage 3. Here, A and B represent graphite layers, while α, β, γ and δ represent the potassium layer, as shown in Fig. S12 in SI. The vibrational modes, accounting for this low-frequency Raman shift, are shown in Fig. 4c, d, e. The Daumas–Herold defects disappear by the complete filling of K^+^ and the formation of a single phase of 3D-ordered stage 1 KC_8_ at the end of the intercalation process. The stage 3 structure is calculated based on both AAA| and ABA| stackings. There is an imaginary mode in the AAA| stacking (Fig. S13, SI), and the eigenvector indicates that the ABA| stacking is indeed a stable structure based on the phonon calculations, in line with the *operando* XRD analysis above. The calculated phonon modes of the ABA| stacking show a low-frequency Raman active mode at a wavenumber of 126 cm^−1^. For the stage 2 structure, these layered materials slide easily, which means it costs very little energy to move in the *ab*-plane. The energy surface of the configurations (AA or AB stacking) is relatively flat. The calculated AαAAαA and AαABαB stacking sequences are both stable, but the AαAAαA stacking sequence has lower total energy, which is consistent with previous work.^15^ The transformation from AB stacking in graphite and stage 3 to AA stacking in stage 2 and stage 1 shows that a high concentration of K^+^ acts to stabilise the graphite layers. The final frequency for stage 1 is 148 cm^−1^, for stage 2 is 138 cm^−1^, and for stage 3 is 126 cm^−1^. The frequency for stage 1 is the highest, and stage 3 is the lowest since higher K^+^ concentration results in higher vibrational energy for graphene layers in the *c*-direction. These values are consistent with the experimental data that the low-frequency signal broadened with increased intensity, which indicated the stage 1 formation (see Fig. 4b). It is worth noting that even at the end of discharge, the low-frequency signal is quite broad, and not only stages 1 but also stage 2 and stage 3 *co-exist*. It is reasonable that some empty interlayers allow the signal from stage 2 and stage 3 to appear, matching that observed for Li-ion intercalation into graphite in the literature.^45^ This phenomenon also agrees with what was observed from the *operando* X-ray and PDF analysis.

Here, we define *d*_1_ as the interlayer distance of graphene layers sandwiching K^+^ and *d*_0_ as the intralayer distance without K^+^ in between (Fig. S12 in SI). For stages 1–3, the value of *d*_1_ is very close, and *d*_0_ is exclusively determined by the stacking, *e.g.*, AA or AB. This means the sandwich (AαA or BαB) is mostly independent of the other layers. The relative positions of K^+^ (noted as α, β, γ, or δ) within graphene layers have a minor effect on the frequency of this Raman active mode. This is due to graphene layers vibrating in opposite directions along the *c*-axis with K^+^ remaining stationary. This indicates the frequency of the mode is dominated by two factors, which are *d*_0_ and *d*_1_. For example, in stage 3 with stacking sequence AαABAαAB (Fig. S14 in SI), the A layers vibrate but the B layers remain stationary since only layers sandwiching K^+^ vibrate. The frequency of the low-frequency Raman active mode is therefore only influenced by *d*_1_ (5.35 Å, A layer distance), and the effect of *d*_0_ (3.35 Å, AB layer distance) is trivial. For stage 2 with AAαAAα stacking sequence, all the A layers vibrate and both *d*_0_ and *d*_1_ influence the frequencies. It has been tested that even an increase of 0.1 Å in *d*_0_ results in a decrease of 10 cm^−1^ in the frequency. For stage 1, since the K^+^ are fully intercalated, all the interlayer distances are defined as *d*_1_, which dominates the frequency of the mode.

### 2.5. Electronic changes

**Fig. 5:**
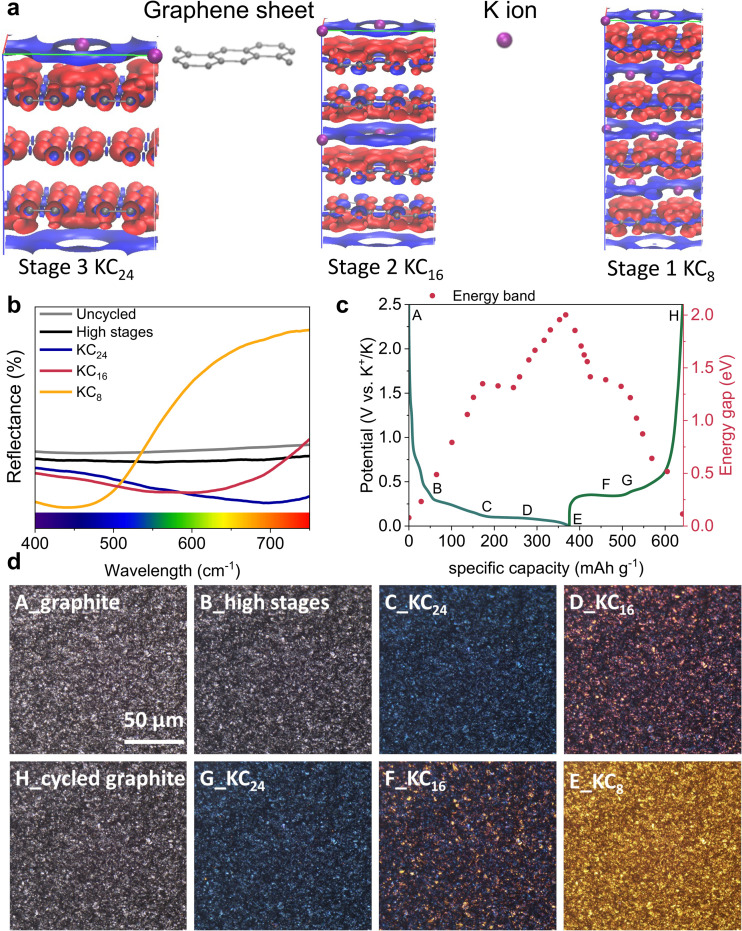
(a) Charge density difference isosurfaces for stage 3, stage 2, and stage 1 compared with the sum of non-interacting pure C and isolated K systems. The isosurfaces are plotted at 0.0012 electrons Å^−3^, with red positive and blue negative; (b) the UV-visible spectrum (visible range of 400 nm to 750 nm) of uncycled graphite, high stages, K_24_ (stage 3), K_16_ (stage 2) and KC_8_ (stage 1); (c) the changes in energy gap (eV) of a graphite electrode during the first potassiation and depotassiation; the A–H are summarised in (d) *operando* microscopy study.

To investigate the electronic structure evolution across stages 3 to 1 K-GICs, DFT simulations were performed using structural parameters derived from XRD and Raman spectroscopy. The changes in electronic structure from stage 3 to stage 1, a DFT simulation on charge difference was performed, based on the structural information provided by XRD and Raman analysis. Fig. 5a and Fig. S15 (SI) visualise the charge density difference for three K-GIC stages, where electron accumulation (red isosurfaces) and depletion (blue isosurfaces) are calculated relative to noninteracting K atoms and pristine graphite. The simulations reveal that potassium intercalation progressively enriches the π orbitals of adjacent carbon rings with electrons. Notably, higher potassium concentrations (*e.g.*, stage 1 KC_8_) correlate with greater electron density in the π system, while unoccupied interlayers exhibit minimal charge redistribution.

Experimental validation is achieved using *operando* UV-Vis differential reflectance spectroscopy (DRS), a technique sensitive to electronic band structure changes. As shown in Fig. 5b, the optical response evolves dynamically during cycling: stage-3 KC_24_ absorbs visible light (500–750 nm), appearing dark blue, and stage-2 KC_16_ shows increased reflectance (650–750 nm), appearing orange, while stage-1 KC_8_ exhibits increased reflectance (400–750 nm), corresponding to green, yellow, and red wavelengths. Kubelka–Munk analysis^56,57^ of the *operando* UV-Vis spectra (Fig. S15) quantifies the energy gap during potassiation/depotassiation (Fig. 5c). The energy gap increases from near 0 eV (pristine graphite) to 2.4 eV (fully intercalated KC_8_) and subsequently decreases during deintercalation. This trend aligns with Fermi level upshifting, which suppresses interband transitions, and only photons exceeding the energy gap are absorbed, while lower-energy light is reflected, explaining the observed colour changes.

In K-GICs, increased potassium concentration delocalises electrons from graphite into the π conduction band (Fig. S16), enhancing metallic behaviour and electronic conductivity,^58^ The same applies to the Li-ion case.^59^ The observed colour evolution of the graphite electrode in Fig. 5d and the Video in SI further corroborates the electronic structure model, as the energy gap modulation directly governs optical properties. This optical behaviour aligns with *operando* UV-Vis DRS and Kubelka–Munk-derived energy gap trends (Fig. 5b and c). The correlation between colour shifts and electronic transitions suggests that simple visible-light monitoring could serve as a cost-effective method to track the status of charge of the graphite anodes in KIBs and beyond. It is noted that some particles can be identified as potassiated (golden colour) while the whole electrode was fully depotassiated, meaning they lose electrical contact with the rest particles or the current collector, leading to a reduction in active material utilisation and also an irreversible expansion of the electrode shown in Fig. 2a.

### 2.6. Surface chemistry

Apart from the structural and electronic changes of graphite, the surface chemistry can also be tuned by forming a passivation layer, the solid electrolyte interphase (SEI) formed on electrode surfaces from the decomposition products of electrolytes.^60^ The degradation of a graphite electrode, caused by large volume change, can lead to particle fracture and loss of network connection,^61^ which can further result in electrode delamination, exfoliation, and vulnerable SEI cracking. Each of these steps contributes to capacity loss by depleting the active potassium inventory of the cell.^34^ XRD and dilatometry analysis both indicate large volumetric variations in both atomic and electrode-level scales. The continuous “breathing” of the graphite particles can cause the SEI layer to crack and rupture, exposing fresh graphite surfaces to the electrolyte. This triggers further electrolyte decomposition and SEI reformation, consuming active potassium and electrolyte, and leading to increased impedance.^10^ Therefore, a robust SEI layer is essential to ensure stable cycling and alleviate battery degradation. The formation of a passivating SEI layer protects the graphite lattice physically, suppresses further decomposition of the electrolyte,^62^ and facilitates the diffusion of ions.^63^ To characterise this layer from a cycled graphite electrode, *ex situ* time-of-flight secondary ion mass spectrometry (ToF-SIMS) was employed.

It is documented that the SEI derived from the most common KPF_6_ electrolyte is the decomposition of solvents, resulting in an organic-rich SEI layer; while the SEI on the graphite electrode with KFSI-based electrolyte is predominantly composed of inorganic compounds, originating from the decomposition of KFSI salt.^13^ Given the increased use of KFSI-based electrolytes in the battery community,^32^ this salt has received more research interest due to its stable SEI and improved cycling performance.

**Fig. 6:**
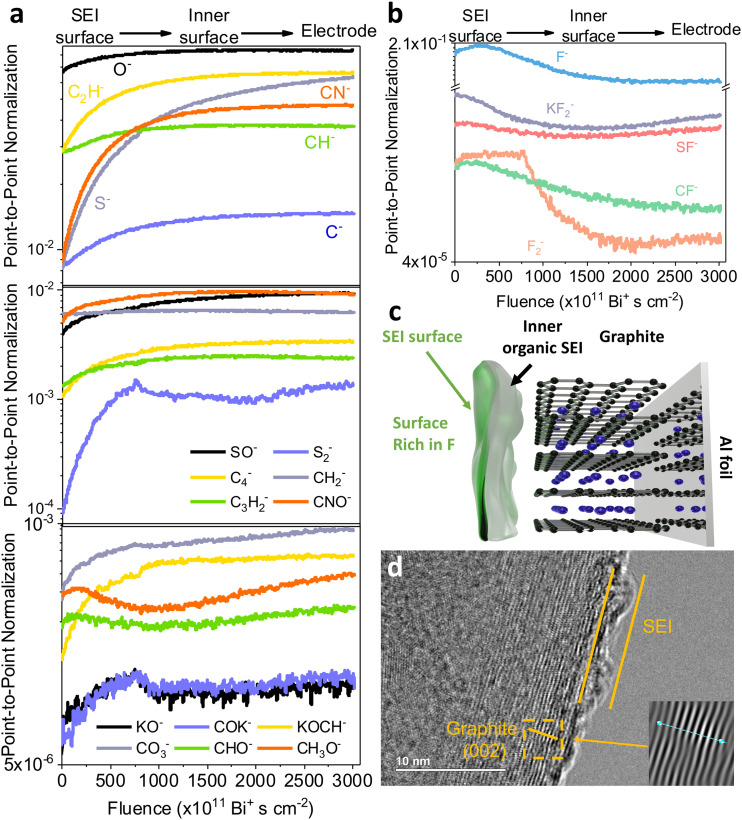
*Ex situ* ToF-SIMS depth profiling (using Bi^+^ primary ion beam only) in the graphite electrode (a) for C-containing, O-containing, and S-containing species, and (b) for F-containing species. All the data are point-to-point normalised to total counts; (c) the schematic diagram of the KFSI-derived thin SEI layer on graphite; (d) an *ex situ* TEM image of the cycled graphite after the first cycle.

According to ToF-SIMS data (Fig. 6a, b, and Fig. S17 in SI), the depth profiles of different species were revealed for both C-containing, O-containing, and S-containing species, and F-containing species. As the SEI layer was thin (with a thickness of 2.0 nm–3.0 nm from Fig. 6c), only a Bi^+^ analysis beam was used to observe the subtle change in the SEI layer. Fig. 6b shows the depth profiles of the SEI layer, focusing on various F-containing species (such as KF^−^, SF^−^, and CF^−^) across the surface, from the surface to the inner SEI layer to the bulk graphite. The analysis revealed a decreasing trend in F-containing species from the surface to the inner phase of the SEI until it reaches a plateau. While it is true that F contaminations are prevalent in most ToF-SIMS analyses conducted in negative mode due to their ubiquity and high electron affinity, we took extra care in our experimental procedures to minimise this effect. Thus, it is unlikely that the high F^−^ signal only results from surface contamination. Instead, it is plausible to deduce that F plays a significant role in the SEI. Moreover, other F-containing species like KF_2_^−^, SF^−^, and CF^−^ provide complementary evidence that a higher concentration of these species is present in the outer SEI compared to the inner layers. These reduction products of the KN(SO_2_F)_2_ salt would be a key factor to demonstrate stable SEIs on the graphite surface.^6^ On the other hand, Fig. 6a shows the depth profiles of the C-containing species such as CO^−^, C_2_H^−^ and CO_3_^−^ across the graphite surface. According to the analysis, C-containing species were concentrated in the inner layer of the SEI. In addition, Fig. 6a shows the depth profile of S-containing species. It is reported that, in KIBs, sulfites could easily accept electrons from the solvents and prevent solvents and K^+^ co-intercalation into the electrode material, in comparison to the oxygen-containing species.^64^ An increase in the concentration of C-containing species and S-containing species can be observed from the surface towards the inner layer until it reaches a plateau where the SEI is sputtered.

It is well-documented that the N(SO_2_F)_2_^−^ anion can result in a robust^65^ but thin SEI layer (less than 10 nm) with an F-containing, specifically KF-rich^66^ protective inorganic layer because of N(SO_2_F)_2_^−^ decomposition^67,68^ It is reported that KF^−^ components can effectively prevent the co-intercalation of solvated K-ions, enabling stable cycling.^69^ In the KFSI in EC/DEC system, it is more likely that the organic phase within SEI is formed by the reduction of the solvent molecules, while S-containing species and F-containing species were formed from anions (N(SO_2_F)_2_^−^). It was hypothesised that the inorganic and organic components represent the flexibility of the SEI layer, while the inorganic elements represent its rigidity.^70,71^Fig. 6c schematically shows the SEI on a graphite anode according to ToF-SIMS analysis, and the structure of the SEI layer is also in good agreement with previous findings.^72^ Especially in KIBs, these flexible organic SEI components can be useful in accommodating the large volume expansion during cycling.^72^ Hence, an organic inner layer with an F-concentrated protective passivated surface could be beneficial to enhance the surface stability for long cycling by preventing excessive depletion of the potassium inventory in the system.

To visualise both the thickness of SEI and the *d*-spacing of the cycled graphite, *ex situ* TEM was also carried out. Fig. 6d shows that the thickness of the as-formed SEI ranges from 2.0 to 3.0 nm after the first cycle compared with the TEM image of pristine graphite (Fig. S18 in SI). The crystallinity of the cycled graphite is inferior to pristine graphite; these findings confirm that K^+^ intercalation disrupts long-range graphite order, favouring disordered lattice rearrangement.

## 3. Conclusion

A series of *operando* characterisation methods was used to elucidate the structural, electronic, and chemical changes induced by electrochemical potassiation/depotassiation in graphite. *Operando* synchrotron XRD and electrochemical dilatometry confirmed the theoretical *c*-axis expansion of 60% at full potassiation from graphite compared to a 49% expansion at the electrode level. Synchrotron XRD and Raman spectroscopy together with DFT calculations provide stacking information as follows: from pristine graphite to stage 1 KC_8_ through liquid-like stages (stage 10L → 9L → ⋯ → 4L) followed by in plane ordered (*p*(2 × 2)*R*0°) stages (KC_24_ stage 3 ABAα, KC_16_ stage 2 AAα) before reaching the final stage 1 KC_8_ (AαAβAγAδ). We also identify the existence of stage 2L between stage 3 and stage 2. UV-vis spectroscopy and optical measurement revealed that the intercalation of potassium leads to an upshifted Fermi level of graphite, as more electrons flow into π orbitals. This change leads to the optical properties, with stage 1 gold KC_8_, orange stage 2 KC_16_ and blue stage 3 KC_24_. *Ex situ* ToF-SIMS explained the superior surface chemistry of the SEI structure, that the outer SEI layer is rich in F-containing species, while the inner SEI layer is more organic. Our results provide a comprehensive fundamental understanding of K^+^ intercalation, revealing the K^+^ storage mechanism in graphite and opening the paths towards further investigation of the K-GICs chemistry.

## Author contributions

Conceptualisation: MT and ZG; supervision: MT; formal analysis: ZG, MT, KW, YZ, C. Molteni, PDH, and FX; funding acquisition: MT; investigation: ZG, YZ, GC, YH, CW, ZS, JJ; methodology: ZG, MT; software (DFT simulation): KW, C. Molteni, and PDH; visualisation: ZG and KZ; writing – original draft: ZG, KW and YZ; writing – review & editing: all authors.

## Conflicts of interest

The authors declare that they have no competing interests.

## Supplementary Material

EB-002-D5EB00184F-s001

EB-002-D5EB00184F-s002

## Data Availability

The authors confirm that the data supporting the findings of this study are available within the article and its supplementary information (SI). The SI incorporates experimental details, graphite characterisation, operando spectra (XRD, Raman, and UV-vis), and simulated K-GIC structures. See DOI: https://doi.org/10.1039/d5eb00184f.

## References

[cit1] EU Batteries , https://environment.ec.europa.eu/topics/waste-and-recycling/batteries_en)

[cit2] Au H., Crespo-Ribadeneyra M., Titirici M. M. (2022). One Earth.

[cit3] Xie F., Niu Y., Zhang Q., Guo Z., Hu Z., Zhou Q., Xu Z., Li Y., Yan R., Lu Y., Titirici M. M., Hu Y. S. (2022). Angew. Chem., Int. Ed..

[cit4] Zhao Y., Kang Y., Wozny J., Lu J., Du H., Li C., Li T., Kang F., Tavajohi N., Li B. (2023). Nat. Rev. Mater..

[cit5] World Economic Forum and Global Battery Aliance, A Vision for a Sustainable Battery Value Chain in 2030: Unlocking the Full Potential to Power Sustainable Development and Climate Change Mitigation, 2019

[cit6] Hosaka T., Kubota K., Hameed A. S., Komaba S. (2020). Chem. Rev..

[cit7] Jian Z., Luo W., Ji X. (2015). J. Am. Chem. Soc..

[cit8] Wu X., Chen Y., Xing Z., Lam C. W. K., Pang S. S., Zhang W., Ju Z. (2019). Adv. Energy Mater..

[cit9] Survey U. S. G. , Mineral commodity summaries 2023: U.S. Geological Survey, U.S. Geological Survey, 2023

[cit10] Zhang W. C., Liu Y. J., Guo Z. P. (2019). Sci. Adv..

[cit11] Liu Y., Shi Y., Gao C., Shi Z., Ding H., Feng Y., He Y., Sha J., Zhou J., Lu B. (2023). Angew. Chem..

[cit12] Min X., Xiao J., Fang M., Wang W. A., Zhao Y., Liu Y., Abdelkader A. M., Xi K., Kumar R. V., Huang Z. (2021). Energy Environ. Sci..

[cit13] Fan L., Ma R., Zhang Q., Jia X., Lu B. (2019). Angew. Chem., Int. Ed..

[cit14] Liu J., Yin T., Tian B., Zhang B., Qian C., Wang Z., Zhang L., Liang P., Chen Z., Yan J., Fan X., Lin J., Chen X., Huang Y., Loh K. P., Shen Z. X. (2019). Adv. Energy Mater..

[cit15] Onuma H., Kubota K., Muratsubaki S., Ota W., Shishkin M., Sato H., Yamashita K., Yasuno S., Komaba S. (2021). J. Mater. Chem. A.

[cit16] Luo W., Wan J., Ozdemir B., Bao W., Chen Y., Dai J., Lin H., Xu Y., Gu F., Barone V., Hu L. (2015). Nano Lett..

[cit17] Li F., Guo Z., Song Z., Wang L., Zheng L., Cheng G., Mattevi C., Hong Z., Titirici M.-M. (2023). Chem. Eng. J..

[cit18] Shi Z., Wang S., Jin Y., Zhao L., Chen S., Yang H., Cui Y., Svanberg R., Tang C., Jiang J. (2023). SusMat.

[cit19] Wei X., Guo Z., Zhao Y., Sun Y., Hankin A., Titirici M. (2025). RSC Sustainability.

[cit20] Abdollahifar M., Doose S., Cavers H., Kwade A. (2023). Adv. Mater. Technol..

[cit21] Zarrabeitia M., Carretero-González J., Leskes M., Adenusi H., Iliev B., Schubert T. J., Passerini S., Castillo-Martinez E. (2023). Energy Mater..

[cit22] Guerard D., Herold A. (1975). Carbon.

[cit23] Yazami R., Touzain P. (1983). J. Power Sources.

[cit24] Fredenhagen K., Cadenbach G. (1926). Z. Anorg. Allg. Chem..

[cit25] Rüdorff W., Schulze E. (1954). Z. Anorg. Allg. Chem..

[cit26] Nishi Y. (2001). Chem. Rec..

[cit27] Clark S. J., Segall M. D., Pickard C. J., Hasnip P. J., Probert M. I., Refson K., Payne M. C. (2005). Z. Kristallogr. – Cryst. Mater..

[cit28] Komaba S., Hasegawa T., Dahbi M., Kubota K. (2015). Electrochem. Commun..

[cit29] Zhang S., Teck A., Guo Z., Xu Z., Titirici M.-M. (2021). Batteries Supercaps.

[cit30] Han H.-B., Zhou S.-S., Zhang D.-J., Feng S.-W., Li L.-F., Liu K., Feng W.-F., Nie J., Li H., Huang X.-J. (2011). J. Power Sources.

[cit31] Michael H., Iacoviello F., Heenan T. M. M., Llewellyn A., Weaving J. S., Jervis R., Brett D. J. L., Shearing P. R. (2021). J. Electrochem. Soc..

[cit32] Wang H., Zhai D., Kang F. (2020). Energy Environ. Sci..

[cit33] Hahn M., Buqa H., Ruch P., Goers D., Spahr M., Ufheil J., Novák P., Kötz R. (2008). Electrochem. Solid-State Lett..

[cit34] Louli A. J., Ellis L. D., Dahn J. R. (2019). Joule.

[cit35] Ziambaras E., Kleis J., Schröder E., Hyldgaard P. (2007). Phys. Rev. B:Condens. Matter Mater. Phys..

[cit36] Diaz-Lopez M., Cutts G. L., Allan P. K., Keeble D. S., Ross A., Pralong V., Spiekermann G., Chater P. A. (2020). J. Synchrotron Radiat..

[cit37] Olsson E., Cottom J., Au H., Guo Z., Jensen A. C. S., Alptekin H., Drew A. J., Titirici M.-M., Cai Q. (2020). Adv. Funct. Mater..

[cit38] Azizi J., Gross A., Euchner H. (2025). ACS Appl. Mater. Interfaces.

[cit39] Yazami R., Reynier Y. (2006). J. Power Sources.

[cit40] Andersen H. L., Djuandhi L., Mittal U., Sharma N. (2021). Adv. Energy Mater..

[cit41] Leung S., Dresselhaus M., Underhill C., Krapchev T., Dresselhaus G., Wuensch B. (1981). Phys. Rev. B:Condens. Matter Mater. Phys..

[cit42] Fujimoto H., Kiuchi H., Takagi S., Shimoda K., Okazaki K.-I., Ogumi Z., Abe T. (2021). J. Electrochem. Soc..

[cit43] Zabel H., Moss S. C., Caswell N., Solin S. A. (1979). Phys. Rev. Lett..

[cit44] Wang Y., Puech P., Gerber I., Pénicaud A. (2014). J. Raman Spectrosc..

[cit45] Yadegari H., Koronfel M. A., Wang K., Thornton D. B., Stephens I. E. L., Molteni C., Haynes P. D., Ryan M. P. (2021). ACS Energy Lett..

[cit46] Baddour-Hadjean R., Pereira-Ramos J. P. (2010). Chem. Rev..

[cit47] Panitz J.-C., Joho F., Novak P. (1999). Appl. Spectrosc..

[cit48] Sole C., Drewett N. E., Hardwick L. J. (2014). Faraday Discuss..

[cit49] Zabel H. and Solin S. A., Graphite intercalation compounds I: Structure and dynamics, Springer Berlin, Heidelberg, 1990

[cit50] Inaba M., Yoshida H., Ogumi Z., Abe T., Mizutani Y., Asano M. (1995). J. Electrochem. Soc..

[cit51] Lui C. H., Heinz T. F. (2013). Phys. Rev. B:Condens. Matter Mater. Phys..

[cit52] Ferrari A. C., Basko D. M. (2013). Nat. Nanotechnol..

[cit53] Giura P., Bonini N., Creff G., Brubach J., Roy P., Lazzeri M. (2012). Phys. Rev. B:Condens. Matter Mater. Phys..

[cit54] Bonini N., Rao R., Rao A. M., Marzari N., Menéndez J. (2008). Phys. Status Solidi B.

[cit55] Bonini N., Lazzeri M., Marzari N., Mauri F. (2007). Phys. Rev. Lett..

[cit56] Landi Jr S., Segundo I. R., Freitas E., Vasilevskiy M., Carneiro J., Tavares C. J. (2022). Solid State Commun..

[cit57] Kubelka P., Munk F. (1931). Zeit. Fer Tekn. Phys..

[cit58] Kang S., Yeom S. J., Lee H. W. (2020). ChemSusChem.

[cit59] Holzwarth N., Rabii S., Girifalco L. (1978). Phys. Rev. B:Condens. Matter Mater. Phys..

[cit60] Wang A. P., Kadam S., Li H., Shi S. Q., Qi Y. (2018). npj Comput. Mater..

[cit61] Gu W., Sun Z., Wei X., Dai H. (2014). Electrochim. Acta.

[cit62] Sonoki H., Matsui M., Imanishi N. (2019). J. Electrochem. Soc..

[cit63] Soto F. A., Marzouk A., El-Mellouhi F., Balbuena P. B. (2018). Chem. Mater..

[cit64] Chen R., Wu F., Li L., Guan Y., Qiu X., Chen S., Li Y., Wu S. (2007). J. Power Sources.

[cit65] Liu P., Ma Q., Fang Z., Ma J., Hu Y.-S., Zhou Z.-B., Li H., Huang X.-J., Chen L.-Q. (2016). Chin. Phys. B.

[cit66] Deng L., Zhang Y., Wang R., Feng M., Niu X., Tan L., Zhu Y. (2019). ACS Appl. Mater. Interfaces.

[cit67] Aurbach D. (2000). J. Power Sources.

[cit68] Howlett P. C., Brack N., Hollenkamp A. F., Forsyth M., Macfarlane D. R. (2006). J. Electrochem. Soc..

[cit69] Qin L., Xiao N., Zheng J., Lei Y., Zhai D., Wu Y. (2019). Adv. Energy Mater..

[cit70] Wang Q., Yang J., Huang X., Zhai Z., Tang J., You J., Shi C., Li W., Dai P., Zheng W. (2022). Adv. Energy Mater..

[cit71] Xia M., Lin M., Liu G., Cheng Y., Jiao T., Fu A., Yang Y., Wang M., Zheng J. (2022). J. Chem. Eng..

[cit72] Wang H., Wang H., Chen S., Zhang B., Yang G., Gao P., Liu J., Fan X., Huang Y., Lin J. (2019). ACS Appl. Energy Mater..

