# Supplementary material for: Revisiting potassium intercalation in graphite: an operando characterisation and computational approach
Source: EES Batter. 2025 Nov 27;2(1):163–75. doi: 10.1039/d5eb00184f (PMC12670375; doi:10.1039/d5eb00184f)
Supplement: EB-002-D5EB00184F-s001 [file EB-002-D5EB00184F-s001.pdf]

Supplementary Information (SI) for EES Batteries.
This journal is © The Royal Society of Chemistry 2025

Supporting Information

Revisiting Potassium Intercalation in Graphite: An *Operando* Characterisation and Computational Approach

Zhenyu Guo[a], Kang Wang[b], Yuanzhu Zhao[a], Gang Cheng[b], Yichen Huang[a], Connor Wright[b], Zonghao Shen[b,c], Hossein Yadegari[b], Jinglin Jiang[a], Fei Xie[d], Kaitian Zheng[a, e], Cecilia Mattevi[b], Carla Molteni[f], Peter D. Haynes[b], Mary P. Ryan[b], and Maria-Magdalena Titirici*[a, g]

[a] *Department of Chemical Engineering, Imperial College London, London, SW7 2AZ, UK. Email: m.titirici@imperial.ac.uk*

[b] *Department of Materials, Imperial College London, London, SW7 2AZ, UK*

[c] *Imperial Global Singapore (IGS), Imperial College London, Singapore, 138602, Singapore*

[d] *Key Laboratory for Renewable Energy, Beijing Key Laboratory for New Energy Materials and Devices, Beijing National Laboratory for Condensed Matter Physics, Institute of Physics, Chinese Academy of Sciences, Beijing, 100190, China*

[e] *Chemical Engineering Research Center, State Key Laboratory of Chemical Engineering, School of Chemical Engineering and Technology, Tianjin University, Tianjin 300072, China*

C. Molteni

[f] *Department of Physics, King's College London, Strand, London, WC2R 2LS, UK*

[g] *Advanced Institute for Materials Research (WPI-AIMR), Tohoku University, 2-1-1 Katahira, Aobaku, Sendai, Miyagi 980-8577, Japan*

Keywords: potassium-ion batteries, graphite, mechanism, operando characterisations, DFT calculations

1. Experimental

1.1 Materials

Synthetic graphite powder was purchased from Alfa Aesar. Poly(vinylidene fluoride) binder (PVDF, average M_w ~ 534000 by gel permeation chromatography, powder), K metal, and CMC binder (Sodium carboxymethyl cellulose, average M_w ~ 250,000, powder) were purchased from Sigma Merck. Potassium bis(fluorosulfonyl)imide (KFSI) (99.9%), diethyl carbonate (DEC, 99.99 %), ethylene carbonate (EC, 99.99 %) solvents were purchased from Guangdong Canrd New Energy Technology Ltd.

1.2 Preparation of graphite electrodes

Graphite anode with CMC binder: a slurry solution of 90 wt.% graphite powder and 10 wt.% CMC solution (pre-prepared, 5 wt.% in Di water) was mixed and stirred overnight. The slurry was then coated onto either Al or Cu foils (9 μm for Cu, 16 μm for Al in thickness), followed by drying in a ventilated oven at 80 ^{o}C overnight before pouching into small electrodes (10 mm in diameter). For *operando* dilatometry and electrochemistry measurements, the mass loading of the graphite active materials is ca. 3 mg cm^{-2}.

Electrode preparation for the synchrotron *Operando* X-ray diffraction

The graphite slurry was prepared by mixing 10 wt.% PVDF (5wt.% in NMP) The graphite electrode was wet coated as 500 μm and, after drying and calendaring, as 120 μm in thickness.to obtain a high thickness of 120 μm after calendaring. This is because the focused X-ray beam size was 50 μm. Then the slurry was coated onto a 250 μm-thick Al foil to keep the coating flat. The thick Al current collector is the key to ensuring the X-ray signal only from carbon. After drying in an 80-degree oven, the electrodes were punched into electrodes with 3mm diameter. Swagelok type cells (DRIX cell) provided by the I15-1 beamline team were used with a thin amorphous quartz tube, with an energy of

70keV and wavelength of 0.161669 Å. The total scattering experiment requires the X-ray beam to pass the active material as much as possible to avoid the effect of either the stainless steel or glass fibre separator/K metal counter electrode. Therefore, a high graphite mass loading (~8-9 mg cm^{-2}) has been coated, and as a result, an extremely low current was applied for potassiation, while a hysteresis was also observed.

1.3 Electrochemistry

Regarding the coin cell assembling, a piece of metallic potassium (12mm in diameter) was used as both the counter electrode and anode. Glassy fibre (Whatman, grade A) was used as the separator, soaked with 100 µL. An argon-filled glovebox (H_2O < 0.5 ppm, O_2 < 0.5 ppm, mBraun) was used to assemble the K-ion coin cells.

1.4 *Operando* dilatometry measurements

The *operando* electrochemical dilatometry (ECD) was performed using a three-electrode ECD-3-nano-aqu (EL-Cell GmbH). A solid and fixed T-frit was used as the separator, ensuring that the thickness change from the working electrode can only be transmitted toward the sensor. Any changes from the metal side will not influence the results. During cell assembling, 400 µL of electrolyte was used for soaking the T-frit, and a separate K metal was used as the reference.

1.5 *Operando* synchrotron X-ray diffraction analysis

Operando synchrotron X-ray diffraction was measured at I15-1 beamline in Diamond Light Source, UK, using a modified Swagelok cell-DRIX cell. Cells were freshly prepared in an argon atmosphere glovebox (O_2 and H_2O < 0.1 ppm) on-site at Diamond Light Source. Electrochemical measurements were performed using an Ivium potentialstat provided by the I15-1 beamline team. The cell was galvanostatically discharged from OCV until the formation of stage

1 K-GICs at 6.5 mA g^{-1}. Due to the limited beamtime available, only the first potassiation till early formation of stage 1 K-GICs was recorded. Measurements were taken using an X-ray beam of energy ~76 keV (λ = 0.161669 Å) and an amorphous silicon area detector (PerkinElmer) to gather data for large values of momentum transfer. Data were collected at 12 min intervals to avoid excessive beam damage, using a total exposure time of 120 s per scan.

1.6 *Operando* Raman Spectroscopy

The configuration of the *operando* Raman cell has been reported before[1,] which was assembled in an Argon-filled glove box (H_2O, O_2 < 0.5 ppm, mBraun). During the measurement, a green laser with a wavelength of 532 nm and 5% power was continuously focused on a fixed graphite particle, and Raman spectra were collected every 600 seconds. To minimise the polarisation of the K metal, an ultralow current (14 mA g^{-1}, 0.05 C) was applied to cycle the cell. During the continuous scanning of the samples, the *operando* Raman cell was simultaneously discharged and charged at a current density of 0.1 C using a LAND CT 3001A battery testing system. The key point is to increase the inner pressure of Raman cells to decrease the polarisation during the discharge and charge testing, which can stabilise the *operando* testing. The configuration of the Raman cell is graphite |1M $KN(SO_2F)_2$ in EC: DEC 1:1 in v.| K metal. The Raman cell was discharged and charged at a constant current of 14 mA g^{-1} (0.05 C) in a voltage window of 1mV-2.5 V.

1.7 Density Functional Theory Calculation Details

Density-functional theory (DFT) calculations are performed using the CASTEP 21.11 package[2] with the PBE exchange and correlation functional.[3] To simulate the van der Waals interactions between the carbon layers, a dispersion correction to the total energy of the system is included using the Grimme scheme[4] as implemented in CASTEP.[5] With the van der Waals correction, the

ionic positions are fully relaxed and then the relaxed structure is used for phonon calculations using density-functional perturbation theory (DFPT).[6] All the calculations are performed with norm-conserving pseudopotentials generated on the fly with a cutoff energy of 1000 eV. The structure relaxation is performed using the Broyden-Fletcher-Goldfarb-Shanno (BFGS) algorithm[7] with force tolerance of 0.001 eV $Å^{-1}$, stress tolerance of 0.001 GPa and a Γ-centered Monkhorst-Pack k-point grid 8×5×2 for K_2C_{48} (stage 3), K_4C_{64} (stage 2) and K_8C_{64} (stage 1).

For the structures in stage 3, stage 2, and stage 1 K-GIC, Previous works[8,9] have identified structures for the different stages; several structures for each stage with various c-axis sequences were characterized at the DFT level. We therefore adopted the lowest-energy structure for each stage and additionally evaluated dynamical stability via phonon calculations. Accordingly, we used AαAβAγAδ for stage-1, AAα for stage-2, and ABA| for stage-3. The ABA| structure was obtained by following the imaginary mode of the reported AAA| stacking sequence

For the choice of supercells, we used three supercells for stage-1, two for stage 2, and one for stage 3, chosen so that the c-axis lattice dimensions are similar across all three stages. Side views of these supercells are shown in Fig.S14.

1.9 *Operando* microscope measurements

In the operando microscope measurement, Celgard was used as the substrate. The graphite electrode was instead coated onto a Celgard film using PVDF as the binder. An optical coin cell with a quartz window (10 mm in diameter and 3mm in thickness) was utilised as the electrochemical cell. The current used in this measurement is 14 mA g^{-1}.

1.10 *Operando* UV-Vis Spectroscopy

In the *operando* optical measurement, the cell configuration remained the same as in the *operando* optical measurements. The graphite electrode was also coated onto Celgard using PVDF as the binder. A Shimadzu UV-2600 instrument with reflectance mode kits was used. The diffuse reflectance mode was used to probe the changes in the colour of the electrode. The wavelength range of the UV-Vis spectrum ranged from 400-1200 nm, the scan speed was selected as “medium”, and the scan interval was selected as 1 nm. Every scan took around 3.5 minutes. The electrode was facing up, thus the colour change can be easily detected by the instrument during electrochemical processes. Before measurement, white powder ($BaSO_4$) was used to calibrate as the measurement background.

1.11 *Ex-situ* Time-of-Flight Secondary Ion Mass Spectrometry

Ex-situ ToF-SIMS electrode preparation: a graphite anode has been cycled at 27.9 mA g^{-1} (0.1 C) for 1 cycle. After charging to 2.5 V, a constant voltage mode was applied for 24h before disassembling in a glovebox with O_2 and H_20 at less than 0.5ppm. The graphite electrode was then washed and soaked with DEC solvent for 6 hours in a glovebox to minimise the salt residue. The air-sensitive samples were prepared and mounted in an Ar-filled glovebox (H_2O < 0.5 ppm, O_2 < 0.5 ppm), and a vacuum transfer suitcase was used to transfer the samples into the load-lock chamber of the instrument. To mitigate the influence of the residue gas in the load-lock pre-chamber, the transfer suitcase was opened until the pressure was lower than $3x10^{-5}$ mbar. During the measurement, charge compensation was achieved with the electron flood gun.

The chemical variation on the solid electrolyte interface (SEI) was depth profiled using the Time-of-Flight Secondary Ion Mass Spectrometry (ToF-SIMS.5, ION-TOF GmbH, Münster, Germany) with a 25 KeV Bi^+ primary ion beam over an

area of 100 x 100 μm^2 in the negative mode. The high current bunch mode (HCBM) of the primary ion beam was applied for the chemical analysis to achieve high sensitivity and mass resolution (~10000). The static mode was applied with the sputtering beam off to minimise the interaction of the sputtering beam with the SEI layers, only with the Bi^+ analysis gun (0.84 pA)

For calculating the dose density (fluence for the x-axis):

analysis time t=5898 s, I (Bi^+)=0.82 pA, Area A=100x100 μm^2 = 10^{-4} cm^2, e = 1.6x10^{-19} C

Primary Ion dose density $PIDD = \frac{It}{eA} = \frac{(0.82 \times 10^{-12}) \times t}{1.6 \times 10^{-19} \times 10^{-4}}$ =0.5125x10^{11} t

Then the sputtering time t can be changed to the corresponding fluence.

So at the end of the measurement, t = 5407s, the total fluence will be

PIDD = 0.5125x10^7x5898 =3.02x10^{14} ions cm^{-2}

1.12 Calculations and equations

The Kubelka–Munk theory, and Planck's Equation (Relationship between light and energy)

$$F(R_\infty) = \frac{(1 - R_\infty)^2}{2R_\infty}$$

$$E = hv$$

E is the energy of the light

v denotes frequency

The theoretical capacity Q_t (mAh g^{-1}) of an electrode material can be determined by Faraday's law as:

$$Q_t = \frac{nF}{3.6\ M}$$

Where n is the number of transferred electrons

F is the Faraday constant (96485 C mol^{-1})

M is the molecular mass of the active material (g mol^{-1}).

In a practical scenario with a constant galvanostatic discharge-charge current, the specific capacity Q_t (mAh g^{-1}) can be calculated as:

$$Q = \frac{I\Delta t}{m}$$

where I (mA) is the discharge/charge current,

Δt (h) is the discharge/charge duration

m (g) is the mass of the active material.

For example, for the stage-1 GIC, with a stoichiometry of KC_8, the molecular mass of C8 is 96 g mol^{-1}, and the number of transferred electrons is 1 as follows:

$$1K^+ + 1e^- + 8C \rightarrow KC_8$$

$$Q_t = \frac{1 \times 96485}{3.6 \ \times 96} = 279.2\ mAh\ g^{-1}$$

For example, for the stage-3 GIC, with a stoichiometry of KC_{32}, the molecular mass of C_{32} is 384 g mol^{-1}, and the number of transferred electrons is 1 as follows:

$$1K^+ + 1e^- + 32C \rightarrow KC_{32}$$

$$Q_t = \frac{1 \times 96485}{3.6 \ \times 384} = 69.8\ mAhg^{-1}$$

Table S1. The stoichiometry of the corresponding theoretical capacity.

Stoichiometry	Stoichiometry	Theoretical capacity (mAh g^{-1})	Theoretical potassiation time under 0.1C °C (hours)
KC_8	$K_{1/8}C$	279.2	10.0
KC_{16}	$K_{1/16}C_8$	139.6	5.0

KC_{24}	$K_{1/24}C_8$	93.1	3.3
KC_{32}	$K_{1/32}C_8$	69.8	2.5
KC_{48}	$K_{1/48}C_8$	46.5	1.7

For stage-n, the average interlayer distance

$$d_{00n} = \frac{5.35 + 3.35 \times (n - 1)}{n}$$

$$d_{00n+1} = \frac{5.35 + 3.35 \times (n - 1)}{n + 1}$$

2. Results and discussion

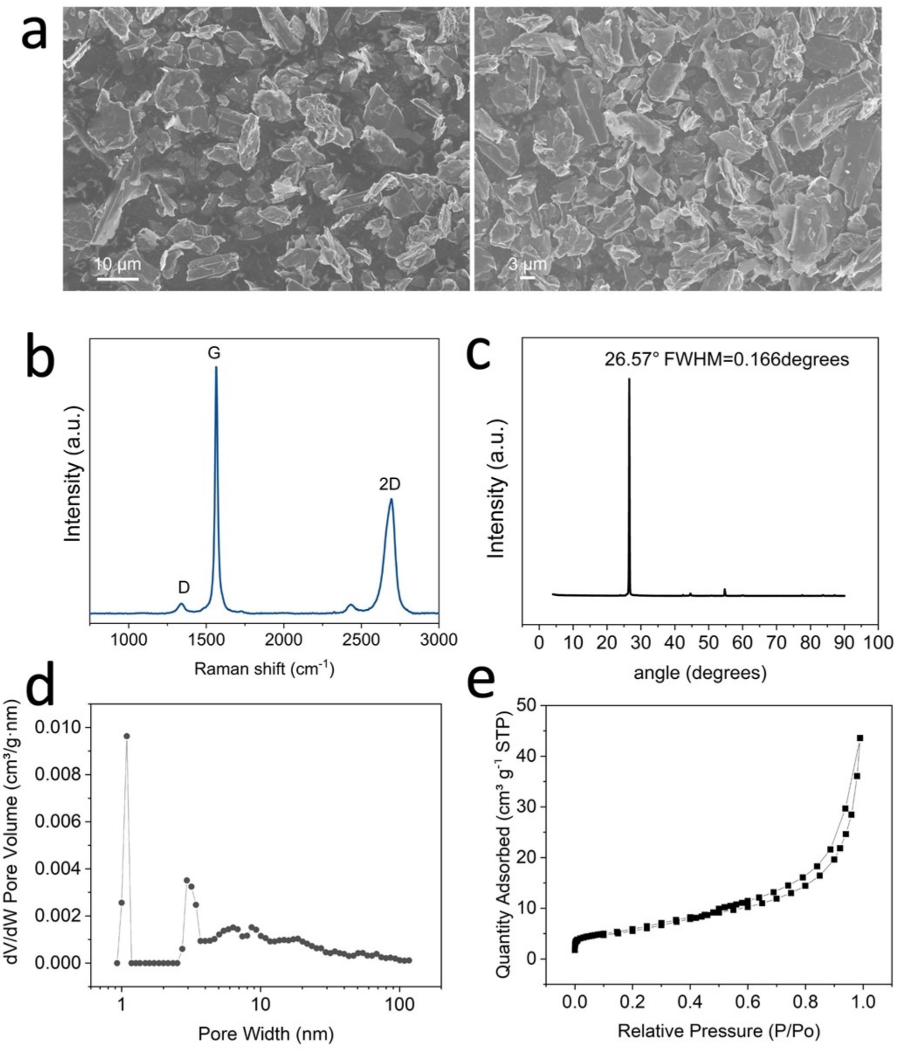

Fig.S1 a) SEM images of pristine graphite; b) The Raman spectrum of the pristine graphite powder, obtained via a 532nm laser. c) The XRD pattern of the as-received pristine graphite powder shows a high degree of graphitisation and strong intensity of the (002) peak located at 26.5 degrees corresponding to the d-spacing of 3.35 Å. d) pore size distribution e) N_2 adsorption/desorption Isotherm at 77K.

The average size of the graphite particle is 7-10 micron. The graphite feature high crystalline structure with negligible d band from Fig.S1b and Fig.1c. The surface area from N_2 BET method is 11.2 m2 g^{-1}.

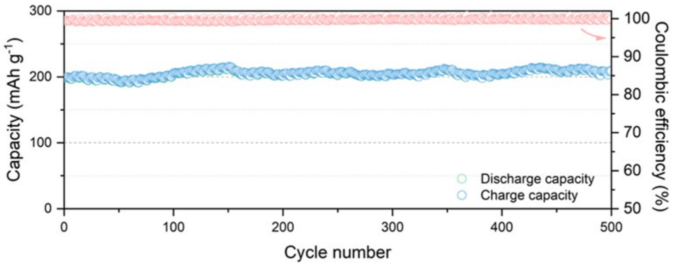

Fig.S2 The long cycling performance from the second cycle of a K||graphite cell using 1M $KN(SO_2F)_2$ in EC/DEC as an electrolyte at a current density of 0.5C (125 mA g^{-1}).

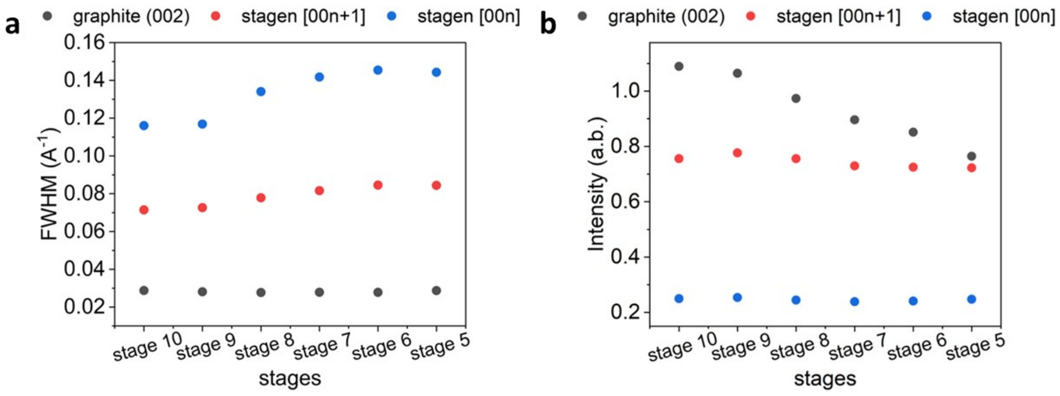

Fig.S3 The peak fitting details summarised a) the FWHM and b) peak intensity from stage 10 to stage 5.

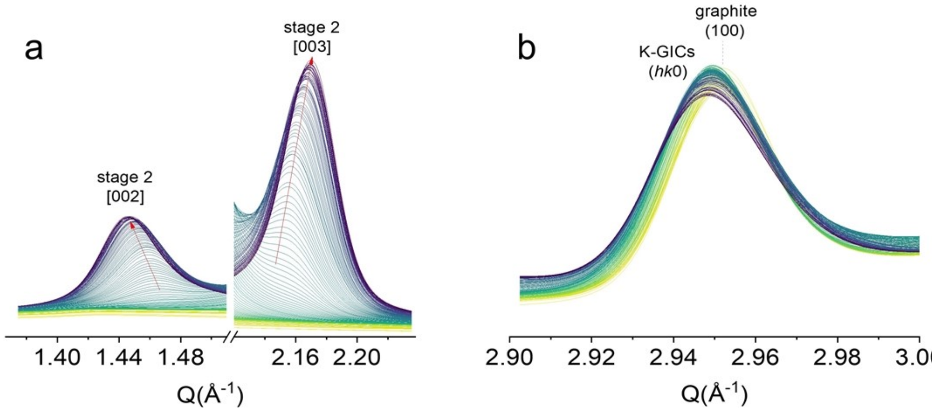

Fig.S4 Operando synchrotron X-ray diffraction patterns for the first potassiation cycle: a) Q range for the formation of [002] and [003] of stage 2 KC_{16}; b) Q range for the graphite (100) peak and (hk0) peaks of K-GICs.

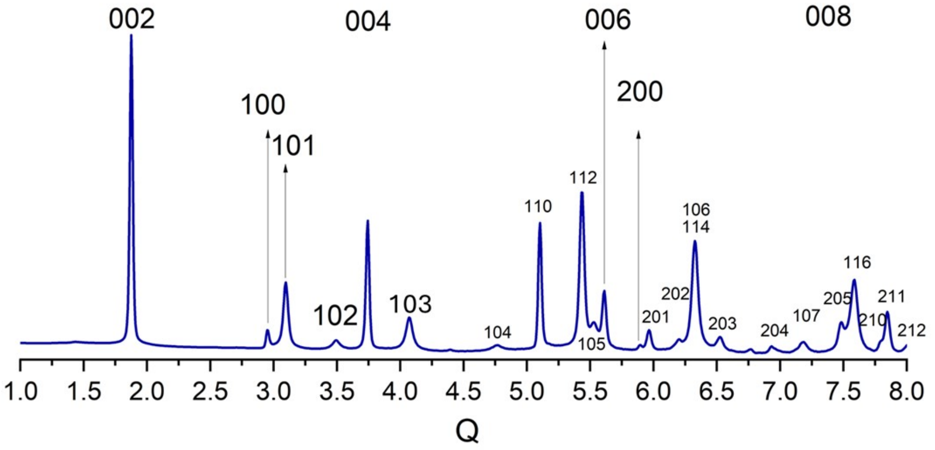

Fig.S5 XRD pattern of the pristine graphite.

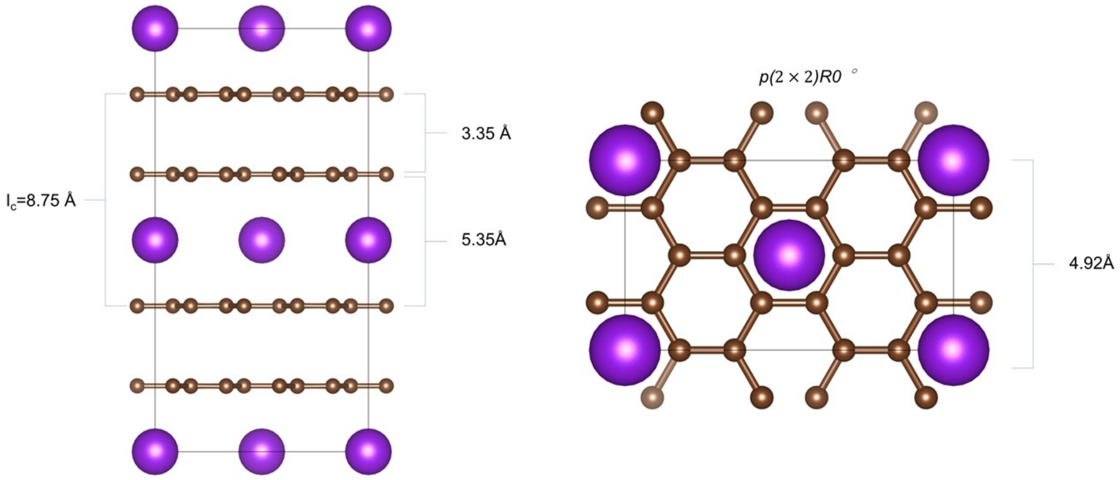

Figure. S6 (left) the side and the (right) top view of the KC_{16} (stage 2 K-GIC).

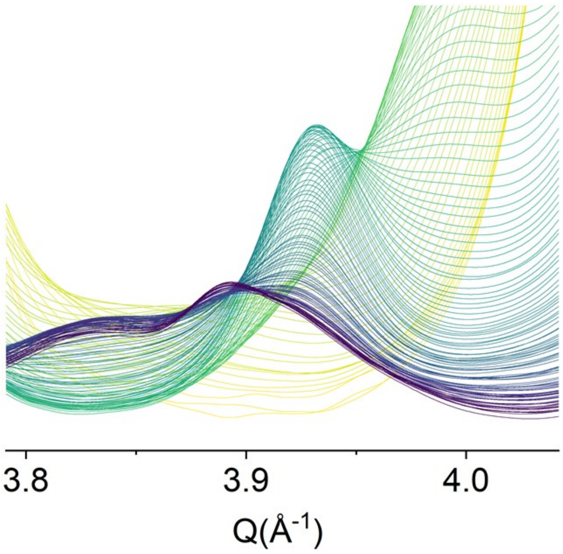

Fig.S7 The (120) in-plane reflection located at ca. 3.88 Å^{-1} of KC_{16} and KC_8

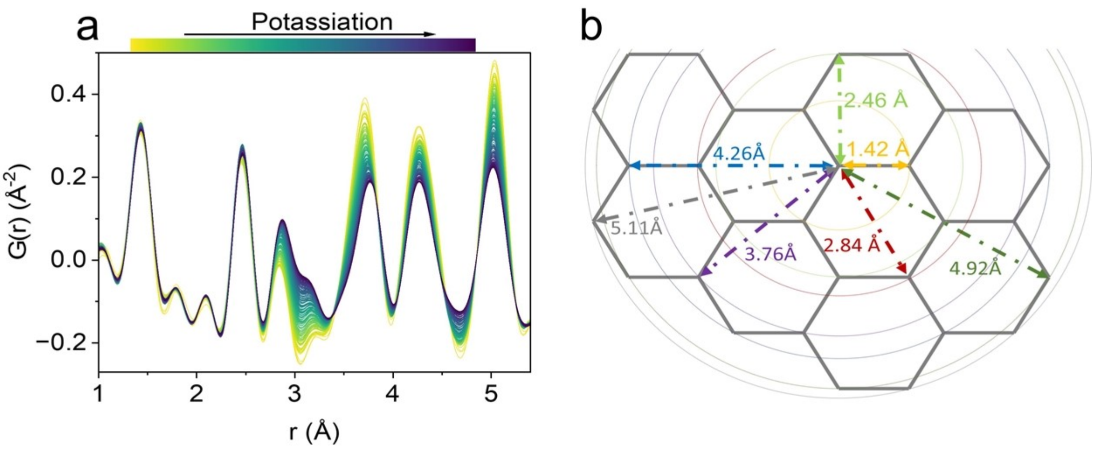

Fig.S8 (a) the operando pair distribution function for the first potassiation cycles; (b) the schematic diagram showing the in-plane distance among carbon atoms.

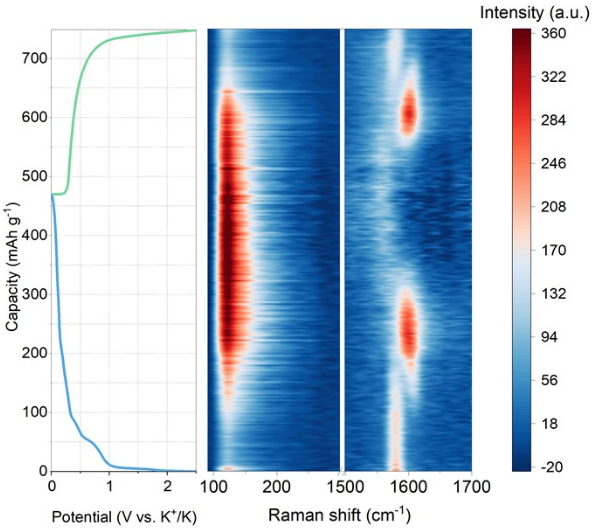

Figure S9. The first discharge-charge cycle in a voltage range of 0V - 2.5 V. Its corresponding *operando* Raman spectra of the low-frequency region and the D band, colored according to their intensity, are shown as a contour plot.

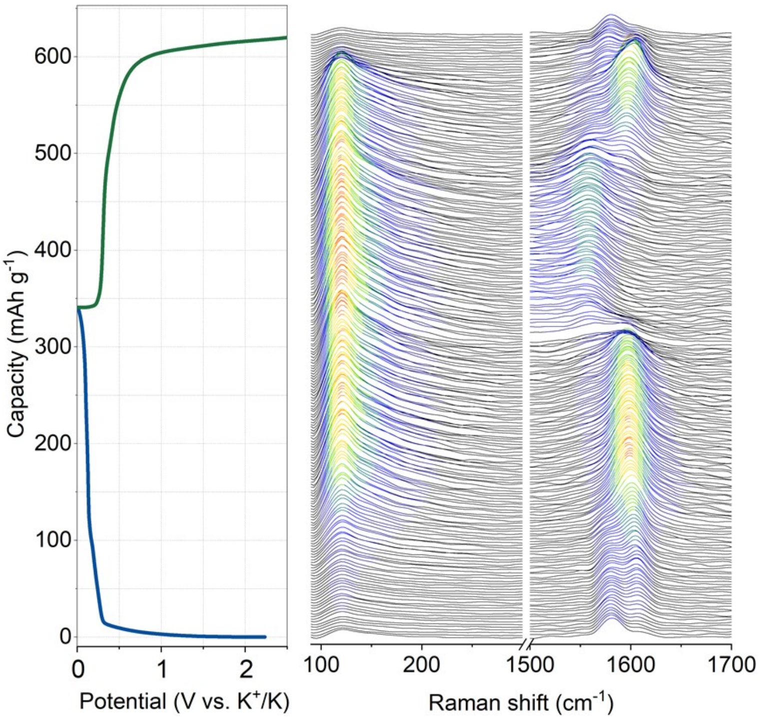

Figure S10. The second discharge-charge cycle in a voltage range of 1 mV – 2.5 V. Its corresponding *operando* Raman spectra of the low-frequency region and the G band, colored according to their intensity, are shown on the right-hand side.

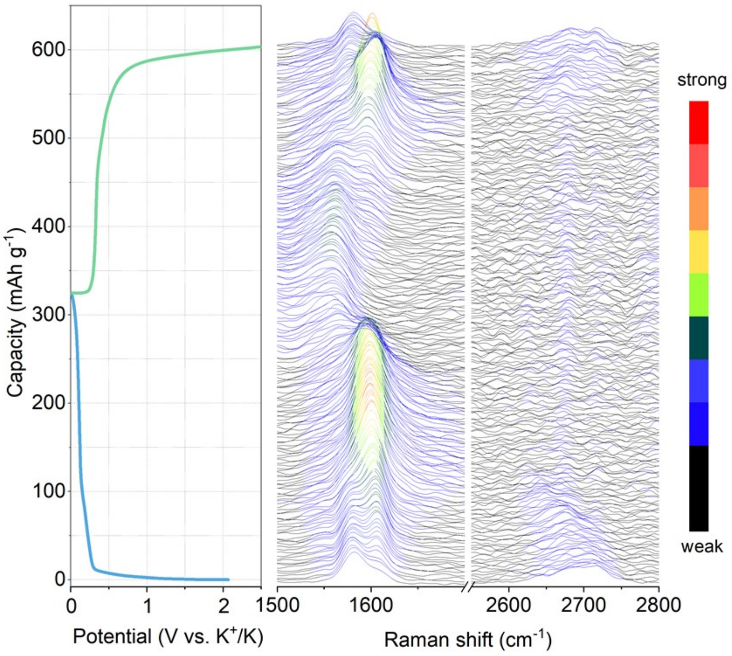

Figure S11. The third discharge-charge cycle in a voltage range of 1 mV – 2.5 V. Its corresponding *operando* Raman spectra of the G band and 2D band, colored according to their intensity.

Figure S10 shows that a red shift of the 2D band (shifted to lower wavenumber) can be observed during potassiation, whereas a blue-shift trend occurs during de-potassiation. The red shift in the 2D band position is caused by both electronic doping and the increase in-plane lattice strain induced by an increasing amount of charge carriers and vice versa. The distorted and disordered graphene layers suggest that the K^+ intercalation is more likely to follow the Daumas-Herold model. Namely, the graphene layers are not fixed but are flexibly deformable around the intercalated ion.[10] For large intercalants, the Daumas-Herold model is a more rational way to reduce energy.[11]

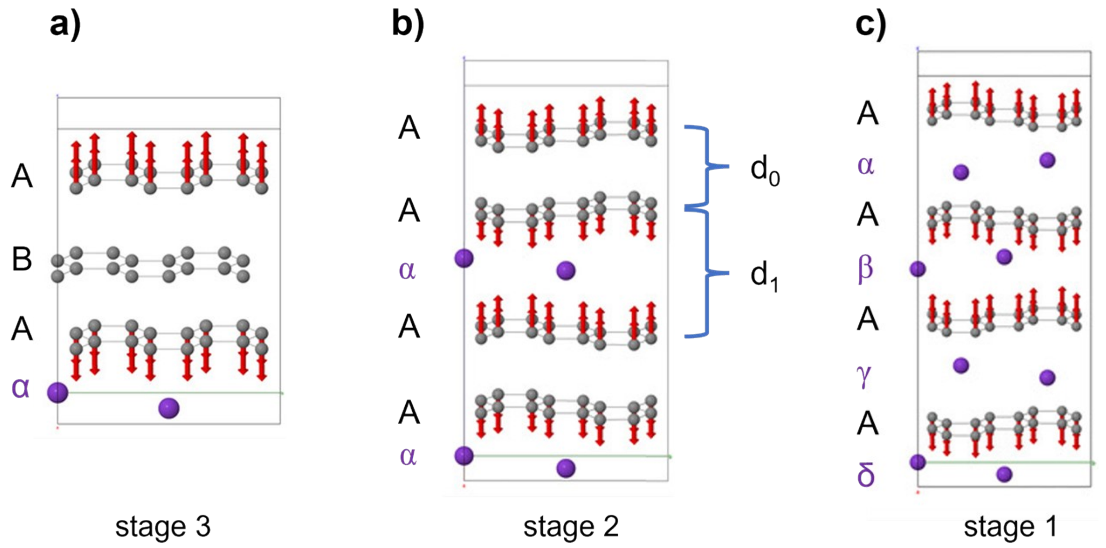

Fig.S12 The corresponding displacement patterns for the Raman-active modes are depicted as a) stage 3, b) stage 2, and c) stage 1 with the stacking information. In Figure S10b, we define d_1 as the interlayer distance of graphene layers sandwiching the potassium layer, and d_0 as the intralayer distance without the potassium layer in between.

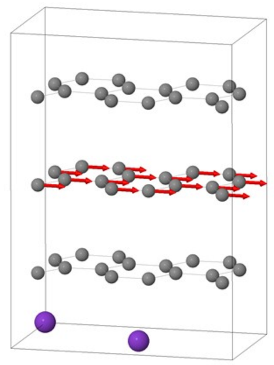

Fig.S13 The displacement pattern of the imaginary Raman active mode calculated for stage 3 stacked in AAA| sequence.

DFT phonon calculations for K_2C_{48} stacked in AAA| sequence resulted in

imaginary frequencies of 37i cm^{-1} at the Γ-point. The displacement pattern of the imaginary frequencies only contains the displacement of the second layer of the C atoms in the b direction, which implies ABA| stacking is more favourable.

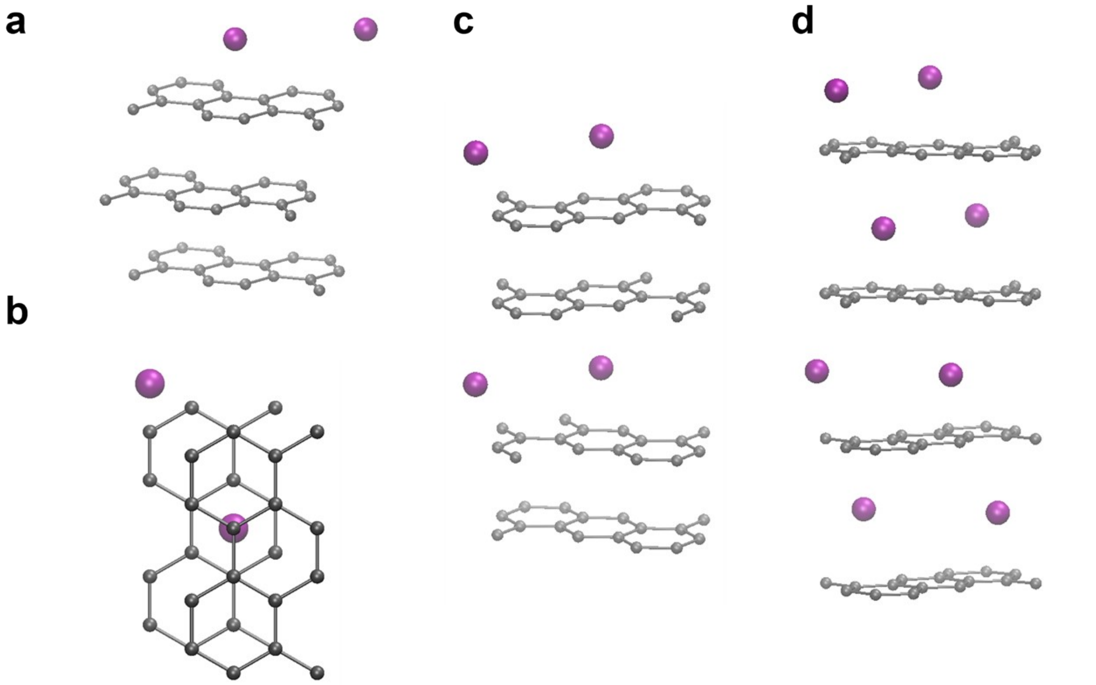

Fig.S14. The structure of different K-GICs: a) stage-3 K-GICs (K_2C_{48}); b) the top view of the stage-3 K-GICs; c) stage-2 K-GICs (K_4C_{64}); d) stage-1 K-GICs (K_8C_{64}).

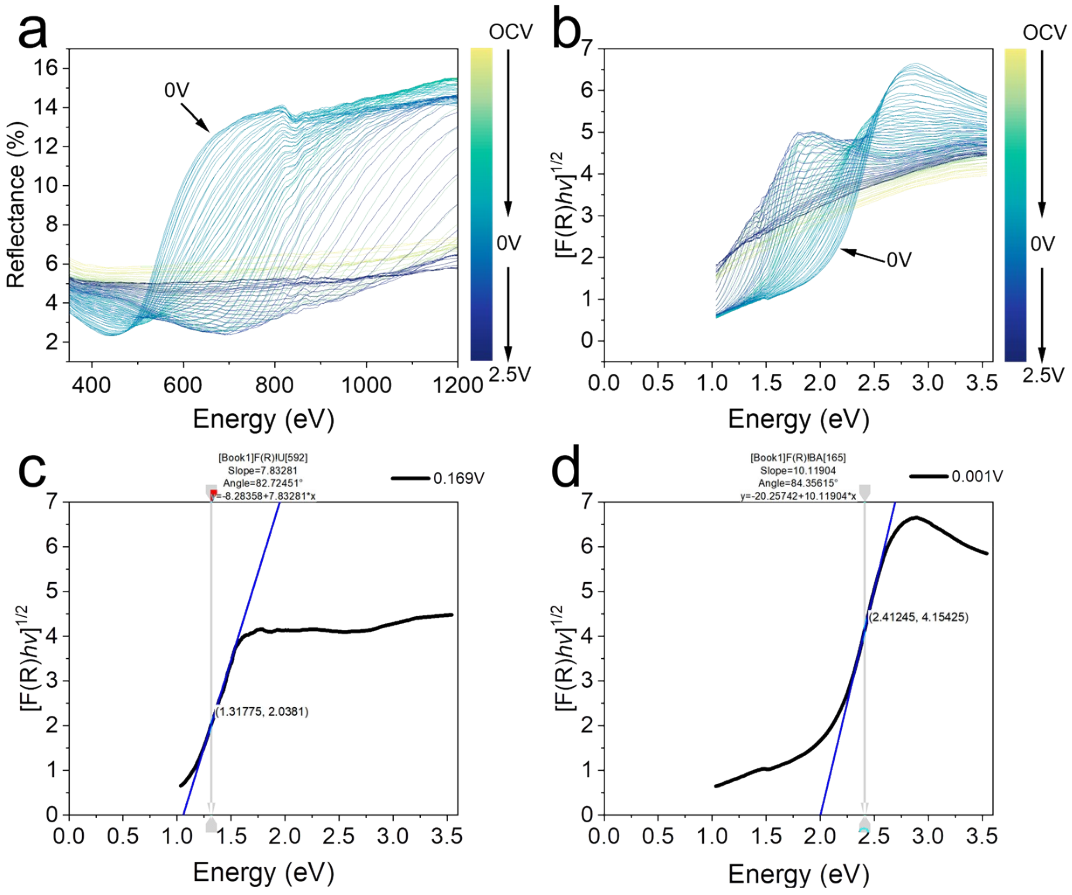

Fig.S15 a) The *operando* UV-Vis spectrum during the first cycle from OCV to 1 mV to 2.5 V, a constant voltage mode was applied after cutoff voltage of 2.5 V was reached; b) By using Kubelka–Munk theory, the UV-vis spectrum data from a) can be expressed as b); c) and d) are representative data treatment.

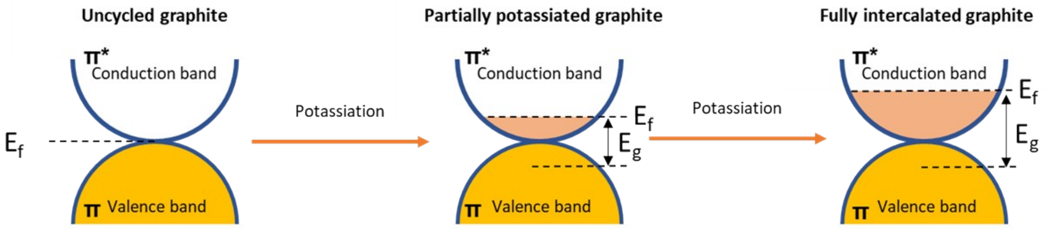

Fig.S16 A schematic diagram of the pi-pi band of graphite, showing how electron was stored in the conduction band of the Pi-Pi band during potassiation. Where E_f is the Fermi level energy, E_g is the interband energy band.

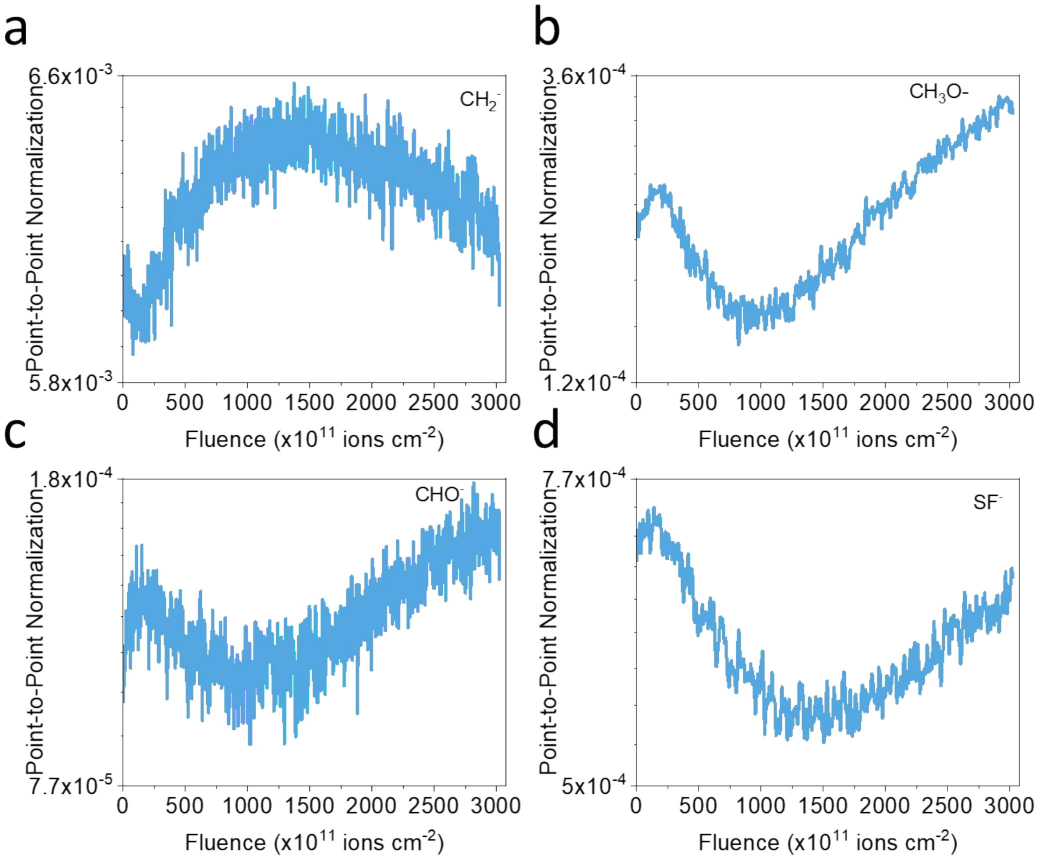

Fig.S17 The individual ToF-SIMS spectrum for a) CH_2^-; b) CH_3O^-; c) CHO^-, and (d) SF^-.

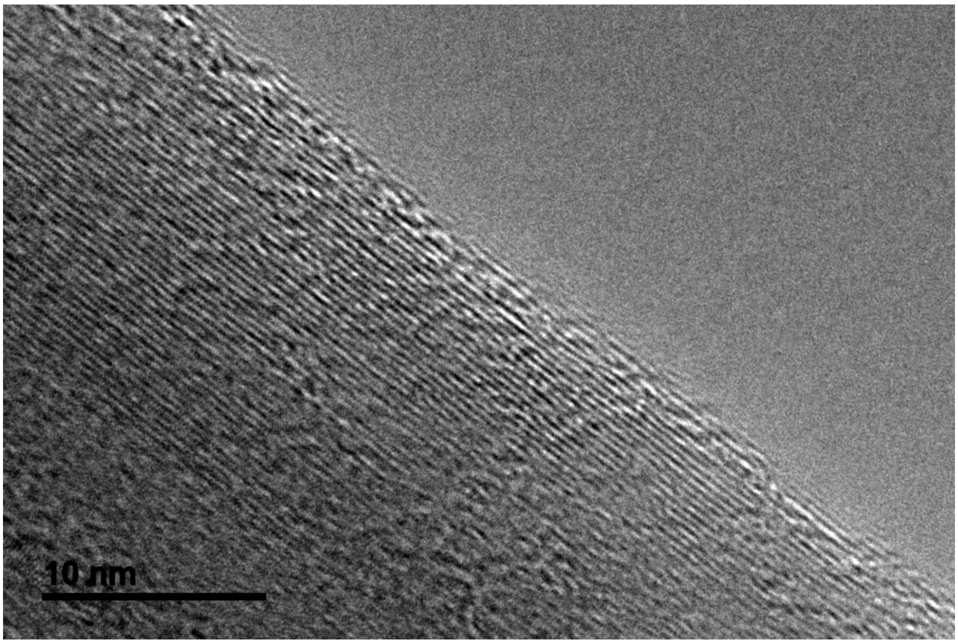

Fig.S18 An HRTEM image of the pristine graphite.

Reference

[1] a) H. Yadegari, M. A. Koronfel, K. Wang, D. B. Thornton, I. E. L. Stephens, C. Molteni, P. D. Haynes, M. P. Ryan, *ACS Energy Lett.* 2021, 6, 1633; b) Z. Xu, J. Wang, Z. Guo, F. Xie, H. Liu, H. Yadegari, M. Tebyetekerwa, M. P. Ryan, Y. S. Hu, M. M. Titirici, *Adv. Energy Mater.* 2022, 12, 2200208; c) Z. Xu, Z. Guo, R. Madhu, F. Xie, R. Chen, J. Wang, M. Tebyetekerwa, Y.-S. Hu, M.-M. Titirici, *Energy Environ. Sci.* 2021, 14, 6381.

[2] S. J. Clark, M. D. Segall, C. J. Pickard, P. J. Hasnip, M. I. Probert, K. Refson, M. C. Payne, *Z Kristallogr Cryst Mater Z KRIST-CRYST MATER* 2005, 220, 567.

[3] J. P. Perdew, K. Burke, M. Ernzerhof, *Phys. Rev. Lett.* 1996, 77, 3865.

[4] S. Grimme, *J. Comput. Chem.* 2006, 27, 1787.

[5] E. R. McNellis, J. Meyer, K. Reuter, *Phys. Rev. B* 2009, 80, 205414.

[6] K. Refson, P. R. Tulip, S. J. Clark, *Phys. Rev. B* 2006, 73, 155114.

[7] J. D. Head, M. C. Zerner, *Chem. Phys. Lett.* 1985, 122, 264.

[8] Z. Jian, W. Luo and X. Ji, J. Am. Chem. Soc., 2015, 137, 11566.

[9] H. Onuma, K. Kubota, S. Muratsubaki, W. Ota, M. Shishkin, H. Sato, K. Yamashita, S. Yasuno and S. Komaba, *J. Mater. Chem. A*, 2021, 9, 11187.

[10] C. Sole, N. E. Drewett, L. J. Hardwick, *Faraday Discuss.* 2014, 172, 223.

[11] a) E. M. Gavilán-Arriazu, O. A. Pinto, B. L. de Mishima, D. Barraco, O. A. Oviedo, E. P. M. Leiva, *Electrochem. commun.* 2018, 93, 133; b) Y. Li, Y. Lu, P. Adelhelm, M. M. Titirici, Y. S. Hu, *Chem. Soc. Rev.* 2019, 48, 4655.
